# Supplementary material for: Morphometric analysis of spread platelets identifies integrin αIIbβ3-specific contractile phenotype
Source: Sci Rep. 2018 Apr 3;8:5428. doi: 10.1038/s41598-018-23684-w (PMC5882949; doi:10.1038/s41598-018-23684-w)
Supplement: Supplementary file 1 — Supplementary Information [file 41598_2018_23684_MOESM1_ESM.pdf]

Supplementary Information for the article “Morphometric analysis of spread platelets identifies integrin $\alpha_{IIb}\beta_3$-specific contractile phenotype”

Sebastian Lickert, Simona Sorrentino, Jan-Dirk Studt, Ohad Medalia,
Viola Vogel, Ingmar Schoen

Contents

1 Supplementary figures 2

2 Additional experimental methods 29
- 2.1 Buffer composition 29
- 2.2 Blood collection and platelet isolation 29
- 2.3 Preparation of poly-acrylamide (PAA) hydrogel substrates 29
- 2.4 Quantification of FG surface density on PAA gel substrates 29
- 2.5 Effect of washing steps before fixation 30
- 2.6 Platelet seeding, sample preparation and fixation 30
- 2.7 Fluorescence stainings 30
- 2.8 Stimulated emission depletion (STED) microscopy 31
- 2.9 Structured illumination microscopy (SIM) 31
- 2.10 Scanning electron microscopy (SEM) 31
- 2.11 Cryo electron tomography (cryo-ET) 31

3 Image analysis 31
- 3.1 Cell outline 32
- 3.2 Actin cytoskeleton 32
  - 3.2.1 Actin fibers 32
  - 3.2.2 Cytoskeletal orientation 33
  - 3.2.3 Radial orientation 33
  - 3.2.4 Calculation of single cell order parameters 34
- 3.3 Vinculin 34
  - 3.3.1 Circumferential profile of adhesions 34
  - 3.3.2 Classification of adhesion morphology 34
  - 3.3.3 Overview plot of adhesion morphology 35
- 3.4 Summary morphology plots of platelet populations 35
  - 3.4.1 Correspondence between vinculin and actin morphological parameters 35
  - 3.4.2 Derived density plots 36
- 3.5 Movies of live cell spreading 36

4 Statistical analysis 37
- 4.1 Box plots 37
- 4.2 Multiple comparisons 37
- 4.3 Comparison of morphology plots 37
- 4.4 Construction of similarity trees 37
- 4.5 Coefficient of variation (CV) 38
- 4.6 Dose-response curves 38

1 Supplementary figures

List of Figures

S1 Live actin dynamics in healthy platelets . . . 3
S2 Image analysis overview . . . 4
S3 Examples of platelet morphologies . . . 5
S4 Density plots of adhesion morphology . . . 6
S5 Mutual dependency of actin parameters . . . 7
S6 Influence of seeding duration on healthy platelets . . . 8
S7 Influence of ADP on morphology of healthy platelets . . . 9
S8 Influence of blood storage duration . . . 10
S9 Donor-to-donor variability . . . 11
S10 Comparison of platelet morphology on different ligands . . . 12
S11 Comparison of platelet morphology with thrombin stimulation . . . 13
S12 DMSO negative control . . . 14
S13 FG surface coverage in ligand titration experiments . . . 15
S14 Blocking efficiency of different surface passivations . . . 16
S15 PAA hydrogel coating efficiency and results . . . 17
S16 dSTORM of healthy platelets . . . 18
S17 Cryo electron tomography . . . 19
S18 Phospho-myosin in healthy platelets . . . 20
S19 Location of the mutation of the GT patient . . . 21
S20 Spreading of platelets from a GT patient is blocked by RUC-4 . . . 22
S21 Morphology of GT platelets: influence of ADP and repeatability . . . 23
S22 dSTORM of GT platelets: actin and vinculin . . . 24
S23 Phospho-myosin in GT platelets . . . 25
S24 dSTORM of GT platelets: microtubuli . . . 26
S25 Influence of seeding duration on platelets from a GT patient . . . 27
S26 Confocal vs. superresolution microscopy methods for imaging F-actin in platelets . . . 28

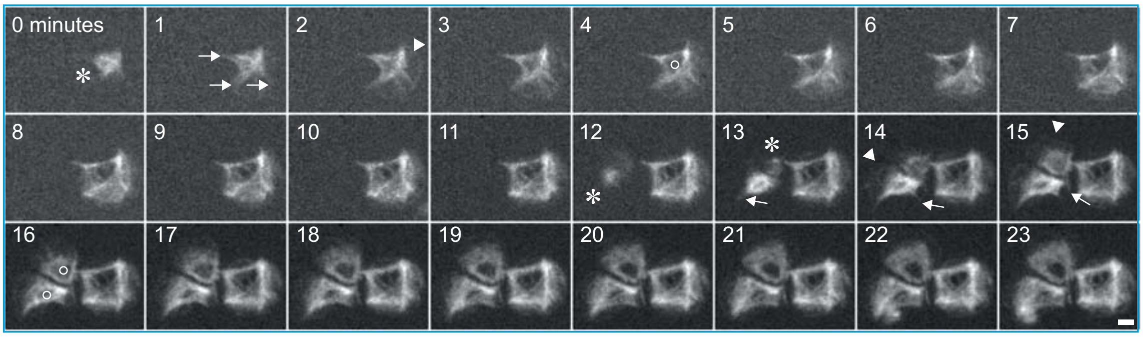

Figure S1: Live cell imaging of the actin cytoskeleton during platelet adhesion on fibrinogen. See also Supplementary Movie S1. Stainings were done by pre-incubating washed platelets for 15 min with the cell permeable dye SiR actin. Asterisks: landing events of single platelets. Arrows: filopodia. Arrowheads: circumferential actin rim. Circle: void middle region, probably containing the granulomer. Scale bar: 2 μm.

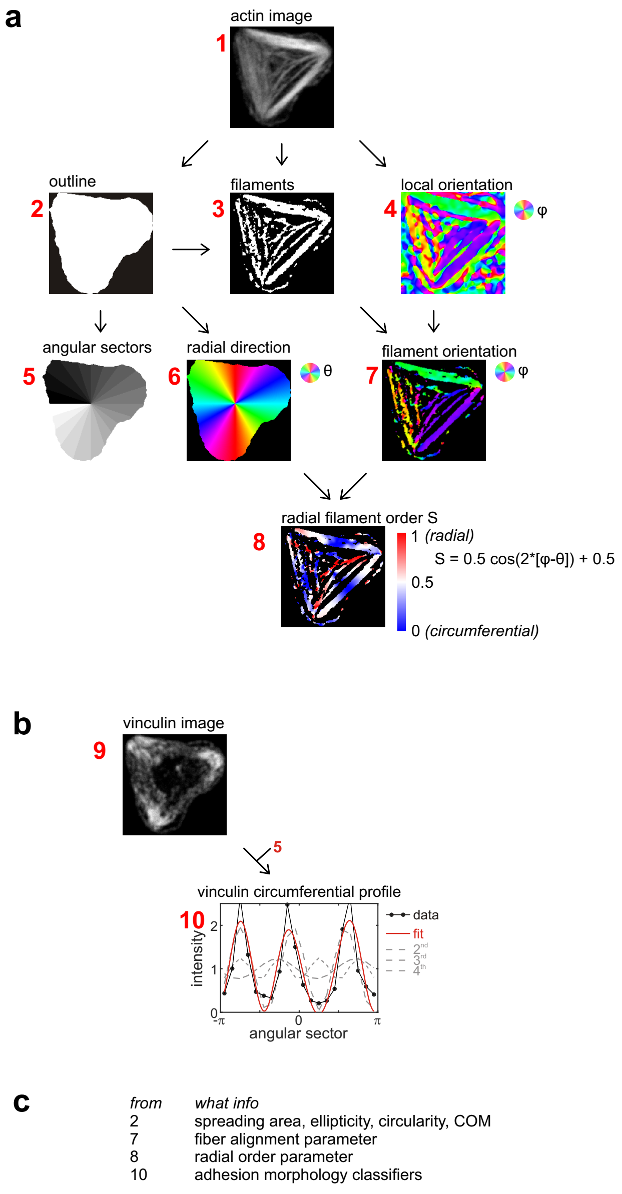

Figure S2: Overview over the image analysis for the quantification of cellular morphology. The steps between images are described in detail in the text. **a)** Operations based on the phalloidin staining of filamentous actin. **b)** Operations using the vinculin staining. **c)** Morphological descriptors extracted from these analyses.

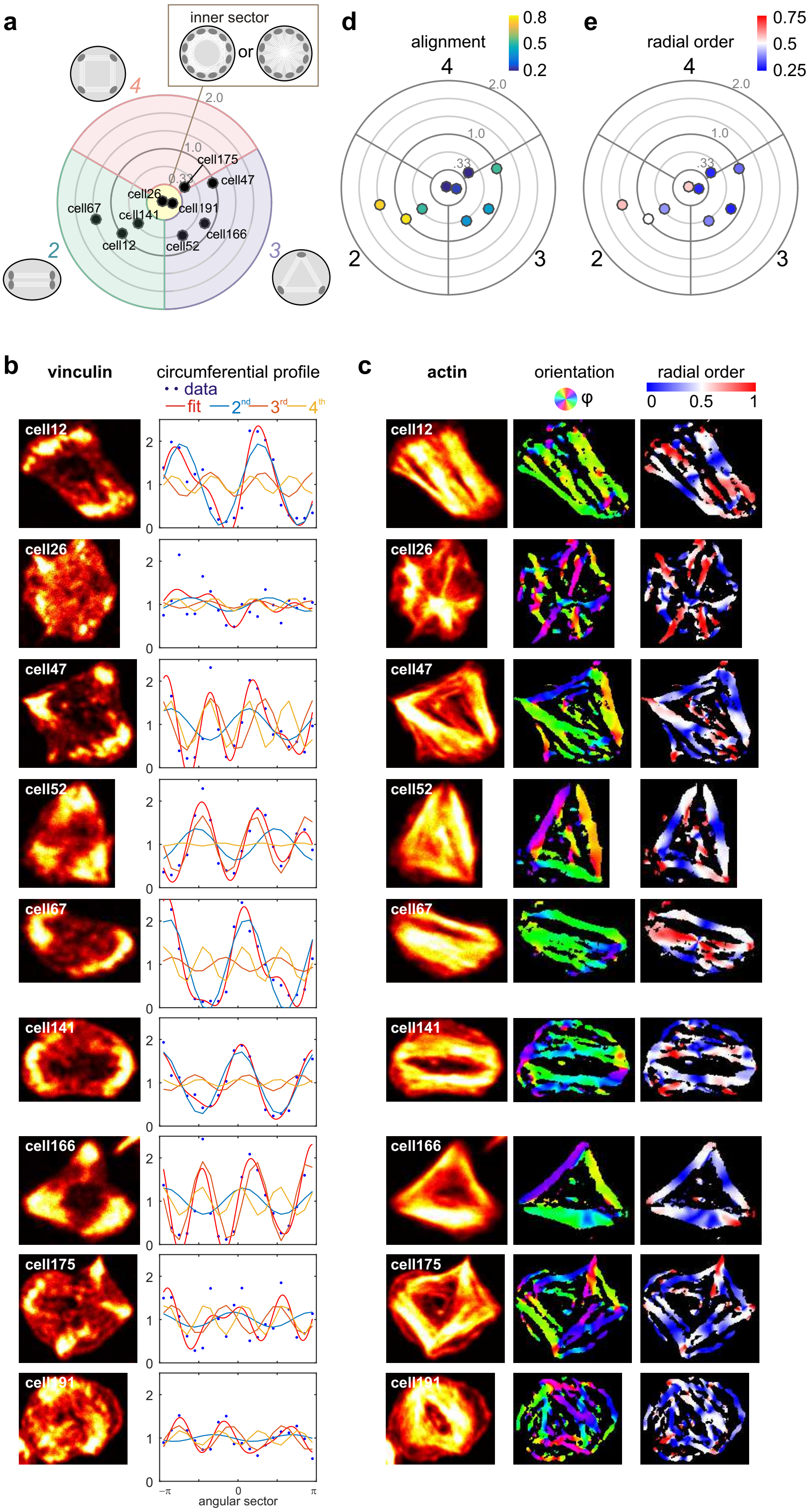

Figure S3: Summarized visualization of cytoskeletal morphology. **a)** Adhesion morphology plot. Each data point designates a single cell; the labels corresponds to the cells in **b+c**. **b)** Morphological analysis of the vinculin staining of single cells. Shown are the vinculin image (left) and the circumferential vinculin profile plus Fourier series fit (right) for the cells. The extracted parameters from the fit determine the placement of each cell in the summary plot in **a**. **c)** Corresponding actin images (left) filament orientation image (middle), and radial order image (right). **d)** Correspondence with actin alignment parameter (color coded). **e)** Correspondence with actin radial order (color coded).

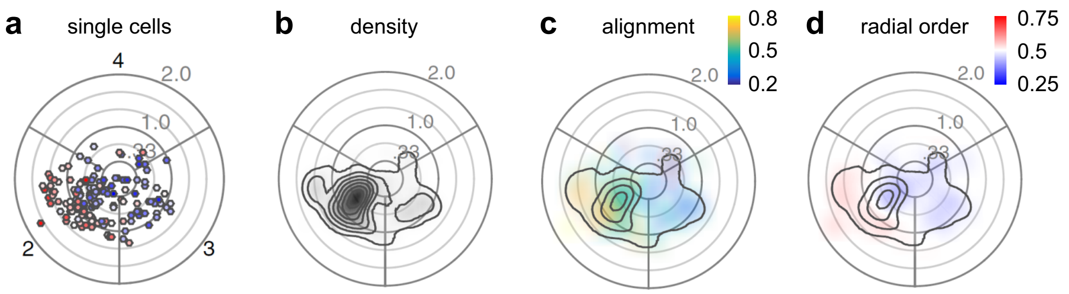

Figure S4: Density plot representations of cytoskeletal morphology. a) Adhesion morphology plot. Each data point designates a single cell. The color coding corresponds to actin radial order. **b)** Contour plot of the cell density of the same data. Contour lines and grayscale color shading refer to the local density. **c)** Overlay of the averaged actin alignment parameter (color-coded) and the contour lines from **b**. **d)** Overlay of the averaged actin radial order parameter (color-coded) and again the same contour lines.

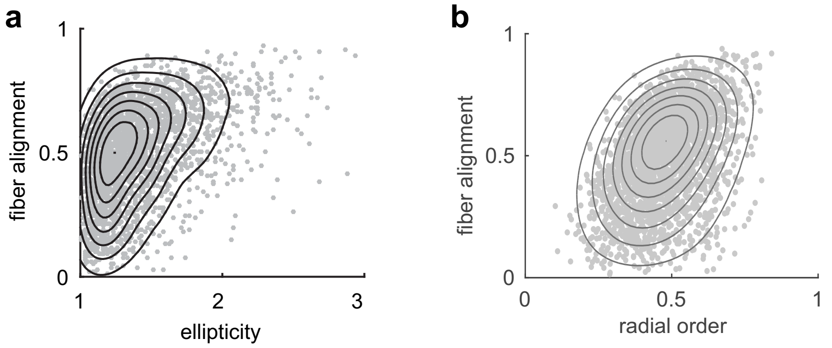

Figure S5: Mutual dependency of actin parameters. a) Correlation between fiber alignment and ellipticity. The data shows that the more elongated the cell, the more aligned are the actin filaments, which is expected. Notable is the high degree of alignment value of approximately 0.35 even for completely round cells (ellipticity=1). This is in contrast to cardiomyocytes that require an aspect ratio of > 3 to align their actin filaments as a pre-requisite to assemble them into sarcomeric structures. **b)** Correlation between fiber alignment and radial order. The fiber alignment and radial order of filaments are not completely independent parameters. Strong fiber alignment favors higher radial order (star-shaped), whereas weak alignment typically goes along with a low radial order (ring-shaped). Note, however, that some cells with weak alignment can show a star-shaped actin arrangement. Data in **a** and **b** from healthy human platelets, same as in Figure 1 **e+f**.

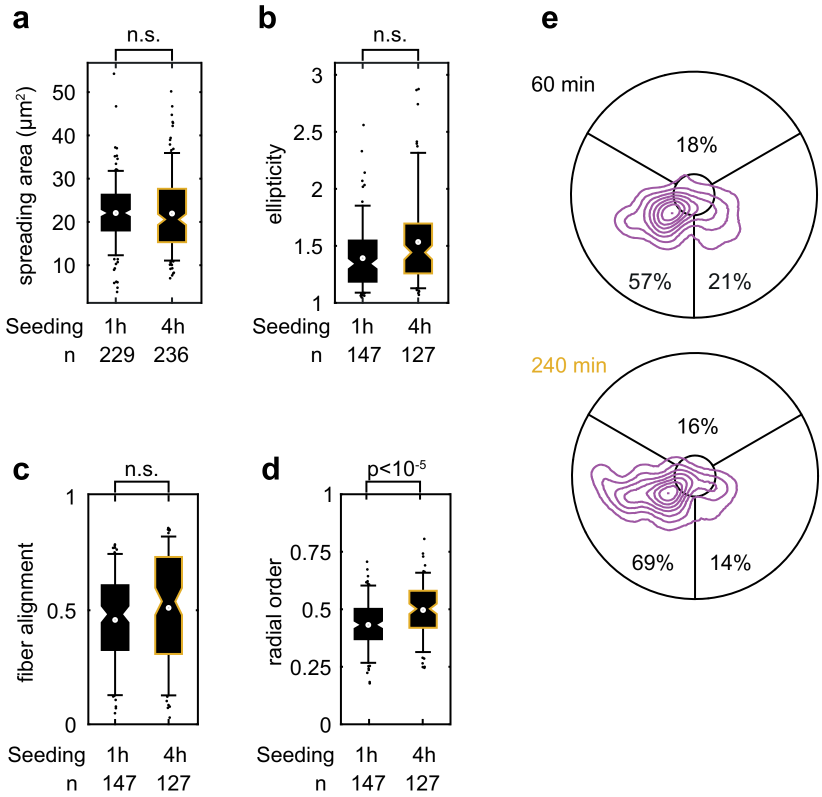

Figure S6: Influence of seeding duration on healthy platelets. Platlets from the same blood sample were seeded for 1 or 4 hours before fixation and staining. The influence on platelet morphology was measured in terms of **a)** spreading area, **b)** ellipticity, **c)** fiber alignment, **d)** radial order, **e)** vinculin adhesion geometry. The polarization of cells slightly increased in terms of their shape, actin fiber alignment and radial order, however, in most cases the changes were statistically not significant. The similarity between vinculin adhesion plots was high with $CC^{\mathrm{morph}} = 0.91$.

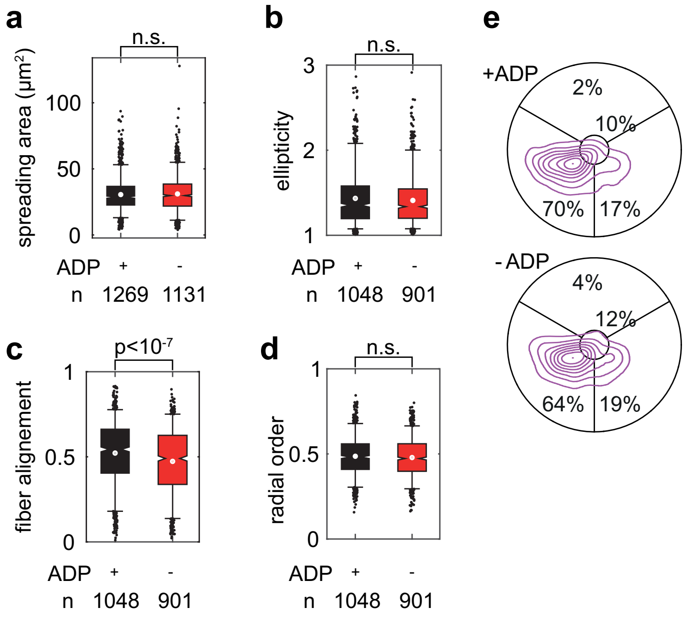

Figure S7: Comparison of platelet spreading in the presence or absence of ADP. Platelet morphology was compared in terms of **a)** spreading area, **b)** ellipticity, **c)** fiber alignment, **d)** radial order, **e)** vinculin adhesion geometry. In the absence of ADP, platelets showed a reduced fiber alignment and less platelets adopted the bipolar morphology whereas spreading area and shape remained unaffected. The similarity between vinculin adhesion plots was moderate with $CC^{\mathrm{morph}} = 0.84$.

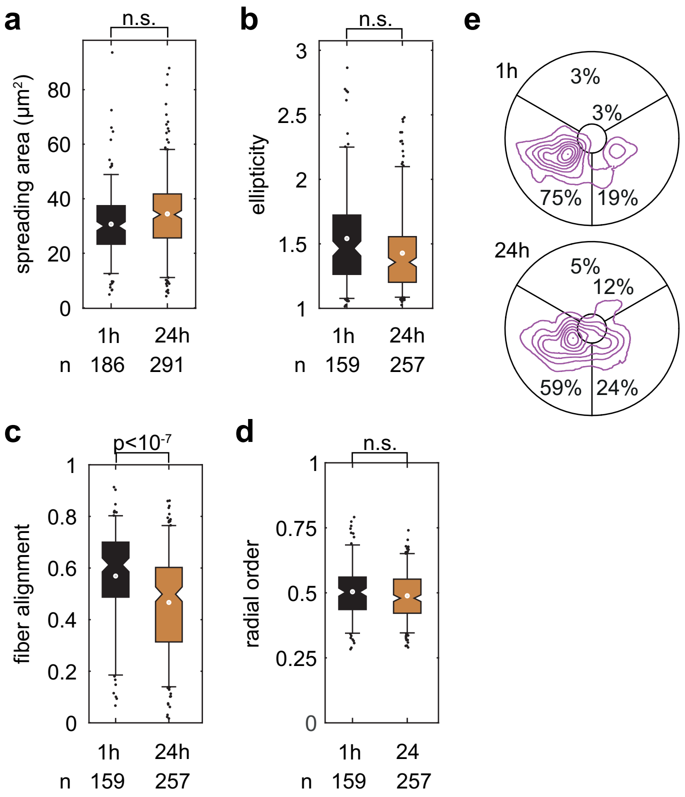

Figure S8: Influence of blood storage duration before assessing platelet spreading. Blood was either directly processed after withdrawal (1h) or one day later (24h). The influence on platelet morphology was measured in terms of **a)** spreading area, **b)** ellipticity, **c)** fiber alignment, **d)** radial order, **e)** vinculin adhesion geometry. After one day storage, the alignment of the actin cytoskeleton was significantly reduced and less platelets adopted the bipolar morphology whereas spreading area and shape remained unaffected. The similarity between vinculin adhesion plots was moderate with $CC^{\mathrm{morph}} = 0.82$.

a

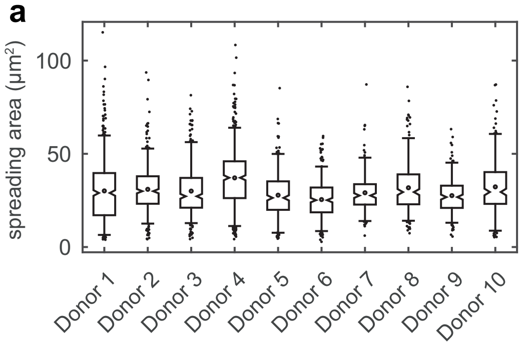

Spreding area (µm²)	Donor 1	Donor 2	Donor 3	Donor 4	Donor 5	Donor 6	Donor 7	Donor 8	Donor 9	Donor 10
Donor 1		n.s.	n.s.	3.65E-12	n.s.	n.s.	n.s.	n.s.	n.s.	n.s.
Donor 2			n.s.	1.40E-05	n.s.	n.s.	n.s.	n.s.	n.s.	n.s.
Donor 3				4.57E-10	n.s.	n.s.	n.s.	n.s.	n.s.	n.s.
Donor 4					7.40E-14	6.60E-25	2.13E-06	n.s.	1.34E-10	n.s.
Donor 5						n.s.	n.s.	n.s.	n.s.	n.s.
Donor 6							n.s.	n.s.	n.s.	n.s.
Donor 7								n.s.	n.s.	n.s.
Donor 8									n.s.	n.s.
Donor 9										n.s.
Donor 10										

b

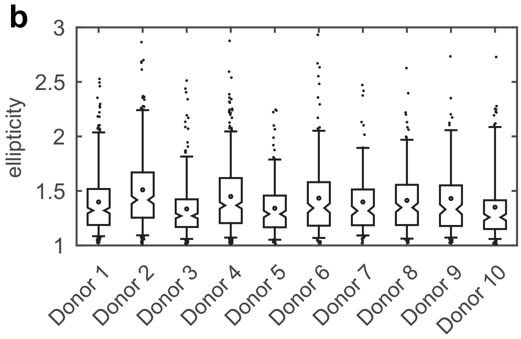

Ellipticity	Donor 1	Donor 2	Donor 3	Donor 4	Donor 5	Donor 6	Donor 7	Donor 8	Donor 9	Donor 10
Donor 1		n.s.	n.s.	n.s.	n.s.	n.s.	n.s.	n.s.	n.s.	n.s.
Donor 2			n.s.	n.s.	6.51E-05	n.s.	n.s.	n.s.	n.s.	4.67E-06
Donor 3				n.s.	n.s.	n.s.	n.s.	n.s.	n.s.	n.s.
Donor 4					n.s.	n.s.	n.s.	n.s.	n.s.	n.s.
Donor 5						n.s.	n.s.	n.s.	n.s.	n.s.
Donor 6							n.s.	n.s.	n.s.	n.s.
Donor 7								n.s.	n.s.	n.s.
Donor 8									n.s.	n.s.
Donor 9										n.s.
Donor 10										

c

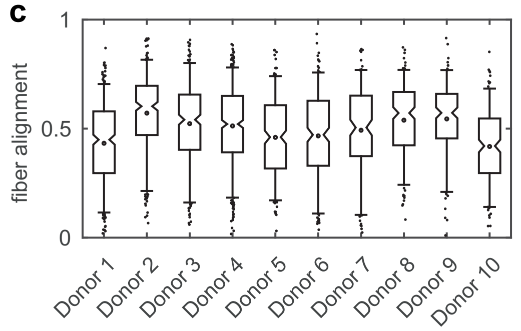

Fiber alignement	Donor 1	Donor 2	Donor 3	Donor 4	Donor 5	Donor 6	Donor 7	Donor 8	Donor 9	Donor 10
Donor 1		2.20E-17	1.44E-05	3.05E-05	n.s.	n.s.	n.s.	3.19E-06	3.84E-06	n.s.
Donor 2			n.s.	n.s.	4.39E-08	5.99E-06	n.s.	n.s.	n.s.	8.14E-15
Donor 3				n.s.	n.s.	n.s.	n.s.	n.s.	n.s.	1.05E-05
Donor 4					n.s.	n.s.	n.s.	n.s.	n.s.	3.25E-05
Donor 5						n.s.	n.s.	n.s.	n.s.	n.s.
Donor 6							n.s.	n.s.	n.s.	n.s.
Donor 7								n.s.	n.s.	n.s.
Donor 8									n.s.	1.71E-06
Donor 9										1.52E-06
Donor 10										

d

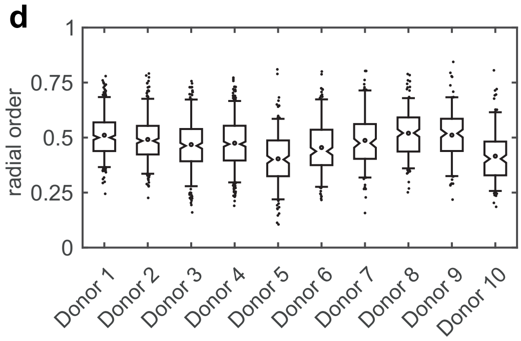

Radial order	Donor 1	Donor 2	Donor 3	Donor 4	Donor 5	Donor 6	Donor 7	Donor 8	Donor 9	Donor 10
Donor 1		n.s.	n.s.	n.s.	2.02E-21	1.87E-05	n.s.	n.s.	n.s.	4.75E-16
Donor 2			n.s.	n.s.	5.52E-12	n.s.	n.s.	n.s.	n.s.	1.41E-08
Donor 3				n.s.	2.76E-05	n.s.	n.s.	n.s.	n.s.	n.s.
Donor 4					1.95E-08	n.s.	n.s.	n.s.	n.s.	1.34E-05
Donor 5						n.s.	5.79E-06	1.11E-18	8.39E-14	n.s.
Donor 6							n.s.	1.99E-05	n.s.	n.s.
Donor 7								n.s.	n.s.	n.s.
Donor 8									n.s.	1.35E-14
Donor 9										9.91E-11
Donor 10										

e

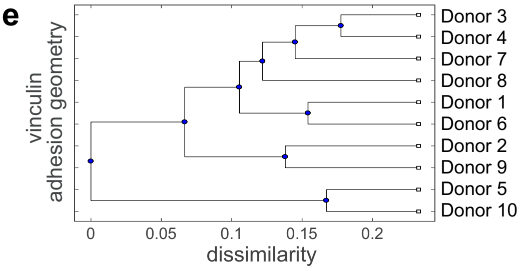

CC^{morph}	Donor 1	Donor 2	Donor 3	Donor 4	Donor 5	Donor 6	Donor 7	Donor 8	Donor 9	Donor 10
Donor 1		0.75	0.92	0.87	0.85	0.92	0.88	0.81	0.79	0.77
Donor 2			0.81	0.83	0.69	0.84	0.84	0.89	0.91	0.56
Donor 3				0.94	0.88	0.90	0.91	0.89	0.83	0.83
Donor 4					0.93	0.87	0.91	0.89	0.87	0.86
Donor 5						0.82	0.85	0.75	0.72	0.93
Donor 6							0.86	0.85	0.84	0.73
Donor 7								0.88	0.84	0.77
Donor 8									0.89	0.67
Donor 9										0.60
Donor 10										

Figure S9: Donor-to-donor variability of platelet adhesion morphology. Shown are boxplot (left) and tables with p-values (right) of ANOVA multiple comparison testing. P-values above 0.1% were considered non-significant. **a)** spreading area, **b)** ellipticity, **c)** fiber alignment, **d)** radial order, **e)** vinculin adhesion distribution geometry. The main morphological characteristics were preserved among platelets from healthy individuals, with the exception of donor 4 (larger spreading area), donor 1 and 10 (reduced fiber alignment), donor 2 (increased fiber alignment and more elongated shape), donor 5 and 10 (lower radial order). This is also depicted by the similarity tree based on vinculin adheison morphology in **e** in which donors 5 and 10 are most dissimilar from the other donors.

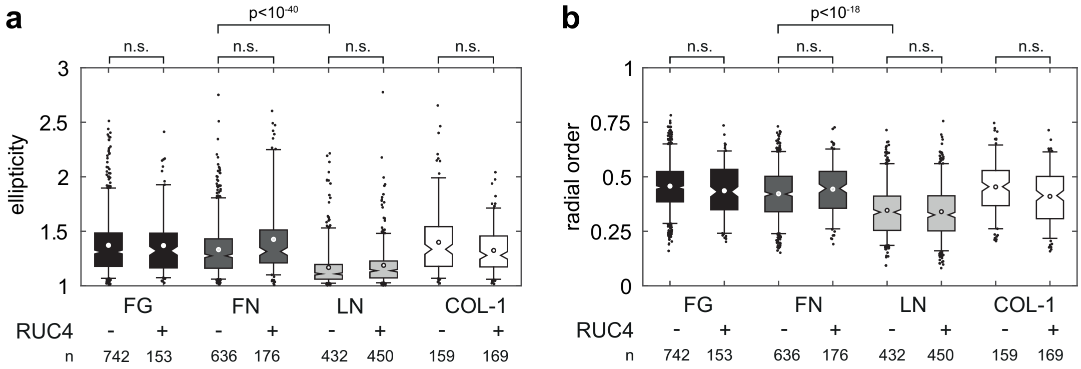

Figure S10: Platelet morphology on FG, FN, LN, and COL1 (cf. Fig.2). Additional morphometrics are **a)** ellipticity and **b)** radial order. Platelets on LN were significantly rounder and had a more circumferential actin cytoskeleton than platelets on FG or FN.

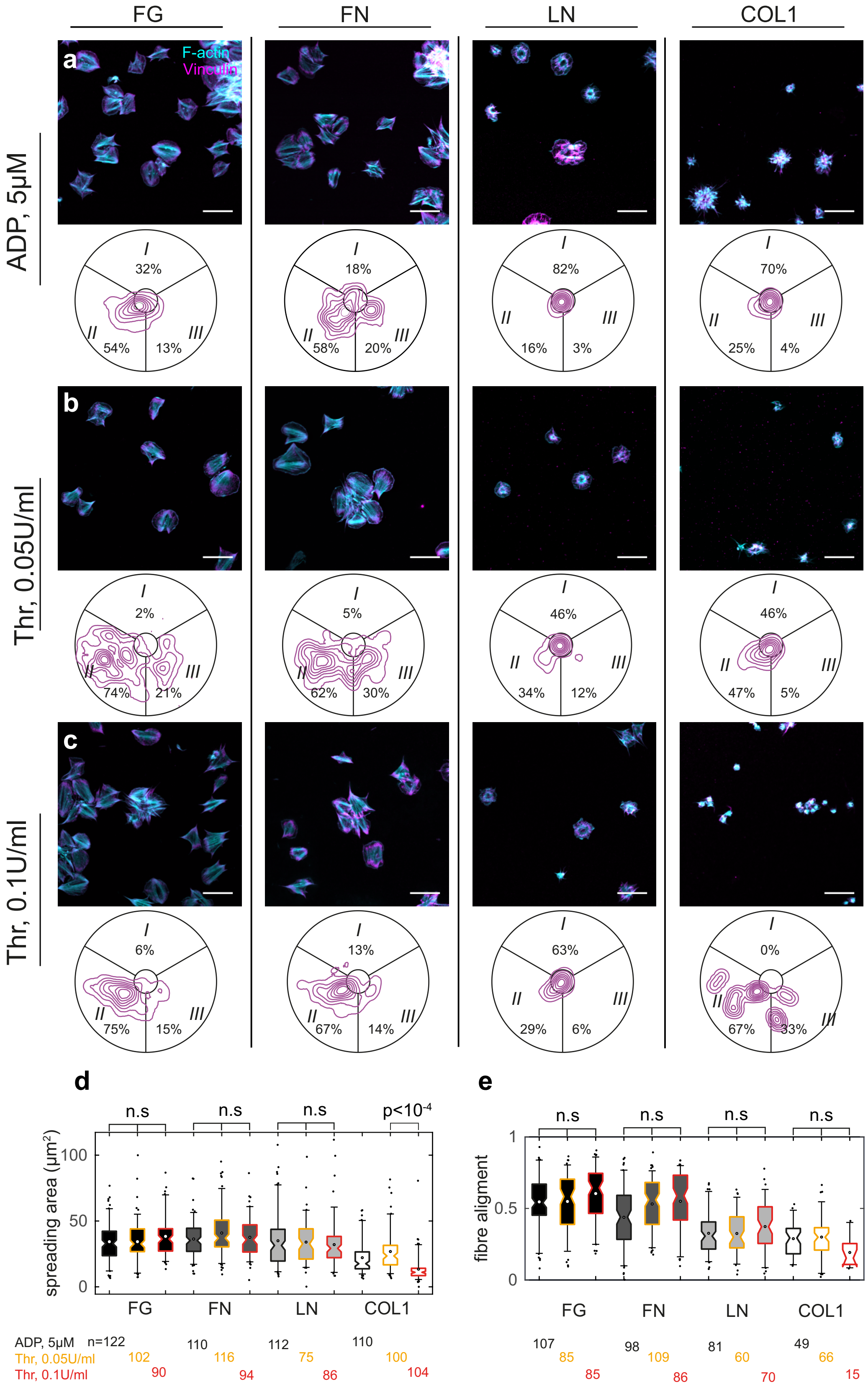

Figure S11: Comparison of platelet morphology on FG, FN, LN, and COL1 with thrombin stimulation. Platelet morphology on FG, FN, LN, and COL1 for platelets that had been seeded in the presence of either **a)** 5 μM ADP, **b)** 0.05 U/mL thrombin (Thr), or **c)** 0.1 U/mL Thr. **a)-c)** show overview images and adhesion site distribution contour plots. Statistical comparisons with regard to **d)** spreading area and **e)** fibre alignment did not reveal statistically significant differences between the different agonists, with the sole exemption for the spreading area on COL1. Data was from one donor only.

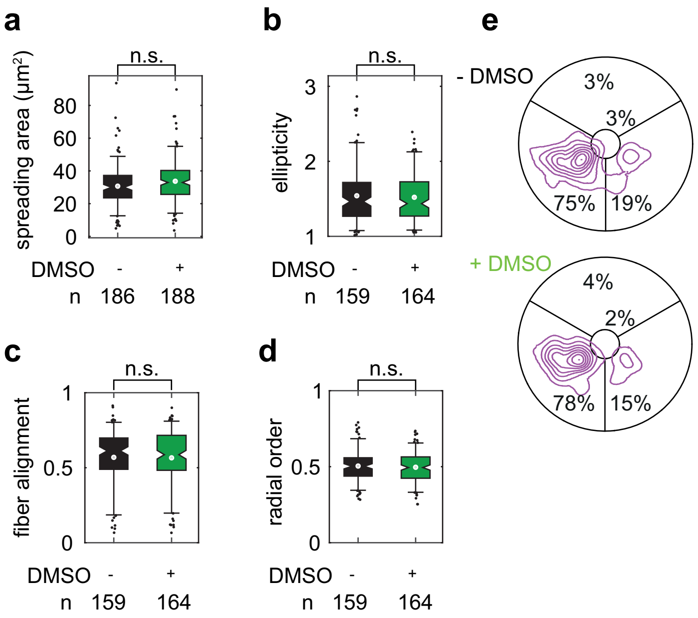

Figure S12: Control experiment with DMSO alone. DMSO was added to the same amount as in the experiments with the integrin inhibitors RUC-4 or eptifibatide. No influence on platelet morphology was observed. The similarity between vinculin adhesion plots was high with $CC^{\mathrm{morph}} = 0.91$.

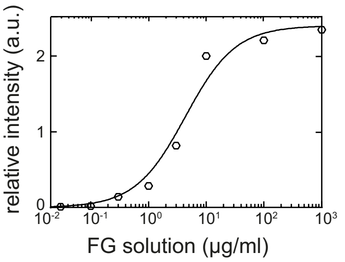

Figure S13: Quantification of adsorbed FG as function of its concentration in solution. The intensity of the fluorescently labeled FG is represented in arbitrary units. The solid line represents a fit with the Langmuir adsorption isotherm. The saturation at high FG concentrations indicates the formation of a monolayer.

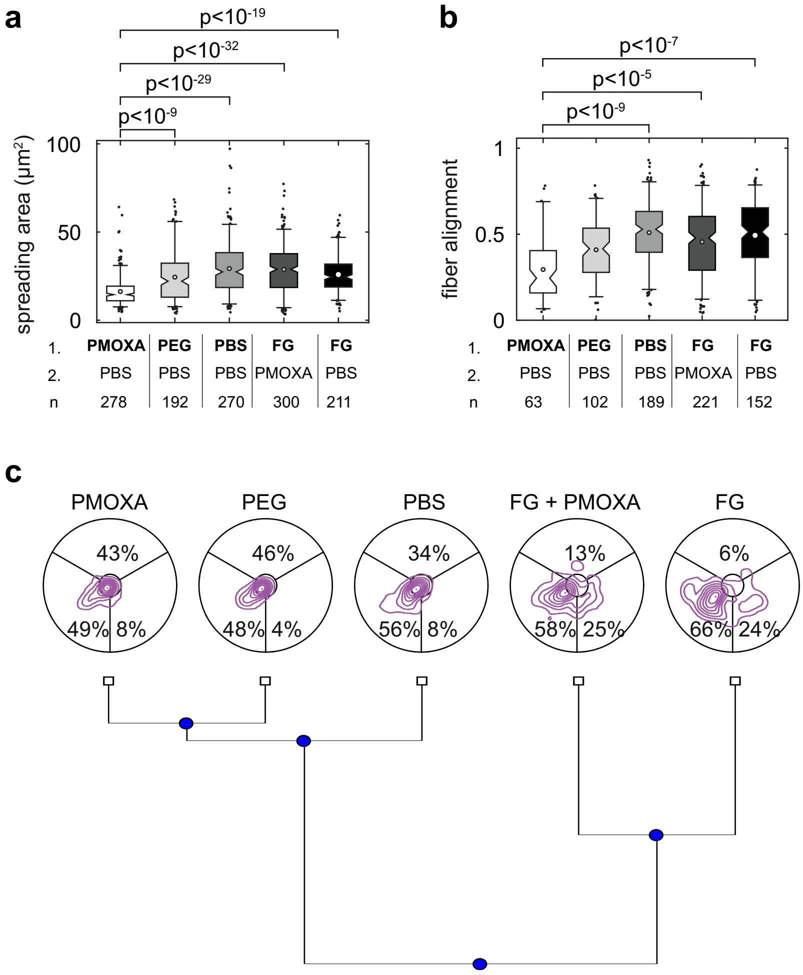

Figure S14: Efficiency of different surface functionalization/passivation procedures. The different procedures comprised FG alone, FG followed by PAcrAm-g-PMOXA, no treatment (PBS alone), PLL-PEG, and PAcrAm-g-PMOXA. Platelet morphology was compared with respect to **a)** spreading area, **b)** fiber alignment, and **c)** vinculin adhesion morphology. While platelets spread normally on FG with a PAcrAm-g-PMOXA post-treatment, passivation by PAcrAm-g-PMOXA completely abolished platelet spreading. A PLL-PEG passivation was less efficient in reducing platelet spreading on the coverslips.

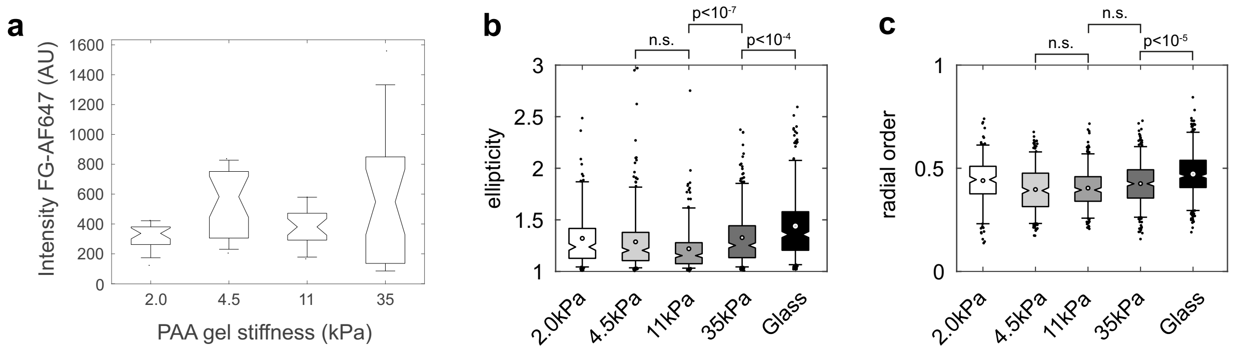

Figure S15: PAA hydrogel coating efficiency and results. a) Quantification of covalently crosslinked fluorescently labeled FG on PAA hydrogels. No systematic or statistically significant differences were observed between gels with 2.0 kPa, 4.5 kPa, 11 kPa, and 35 kPa stiffness. **b)** Ellipticity of healthy platelets on gels. **c)** Radial order of actin cytoskeleton on gels.

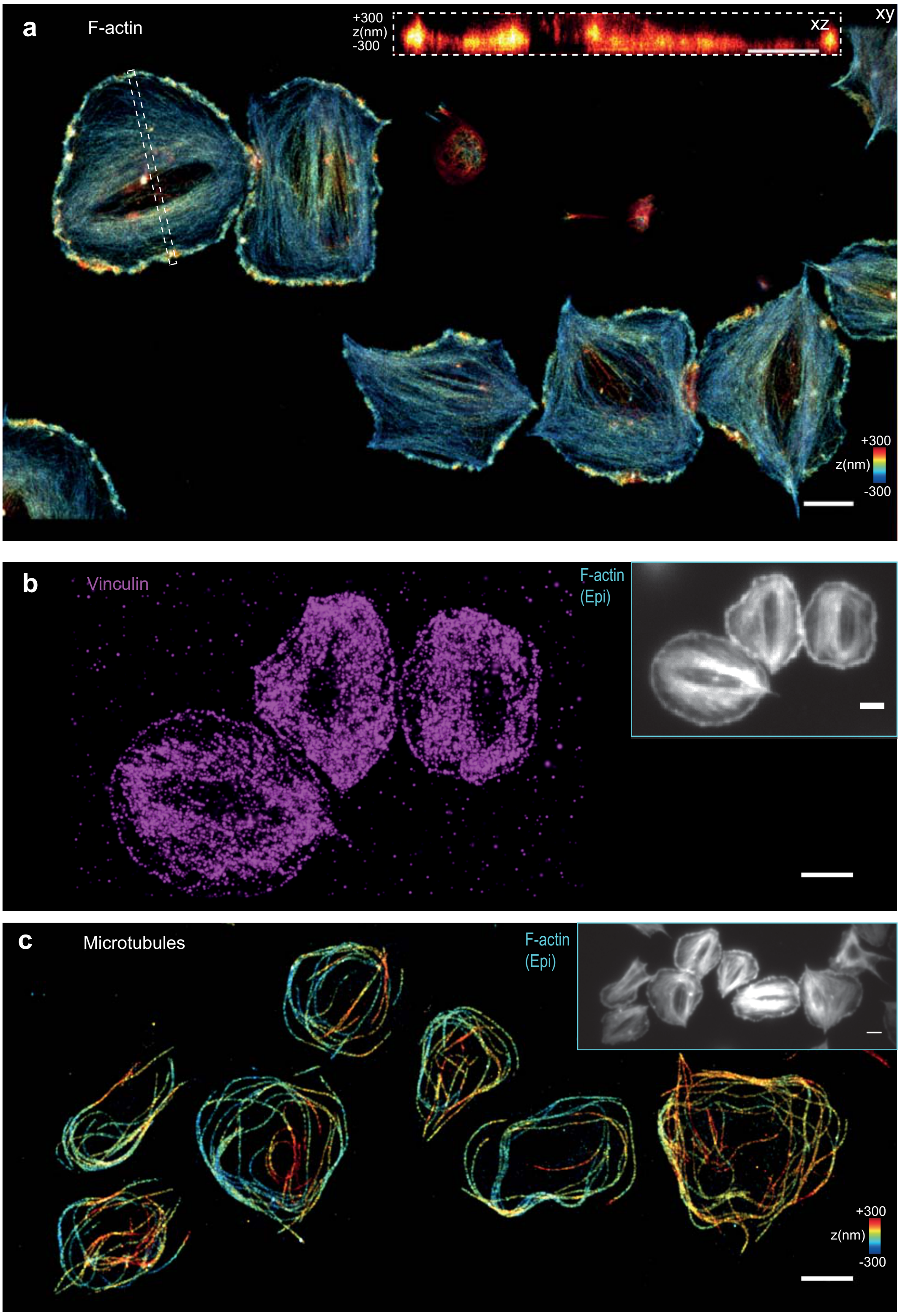

Figure S16: Superresolution (dSTORM) overview images of healthy platelets. a) F-actin is organized in bundles spanning the cell. The inset represents a side view (xz) of the boxed region and demonstrates a very flat (<200 nm) morphology, with the exception of the outer rim and the middle (granulomer) region. **b)** Vinculin forms string-like patterns at the end of actin filaments. **c)** Microtubules are largely uncoiled. Scale bars: 2 μm.

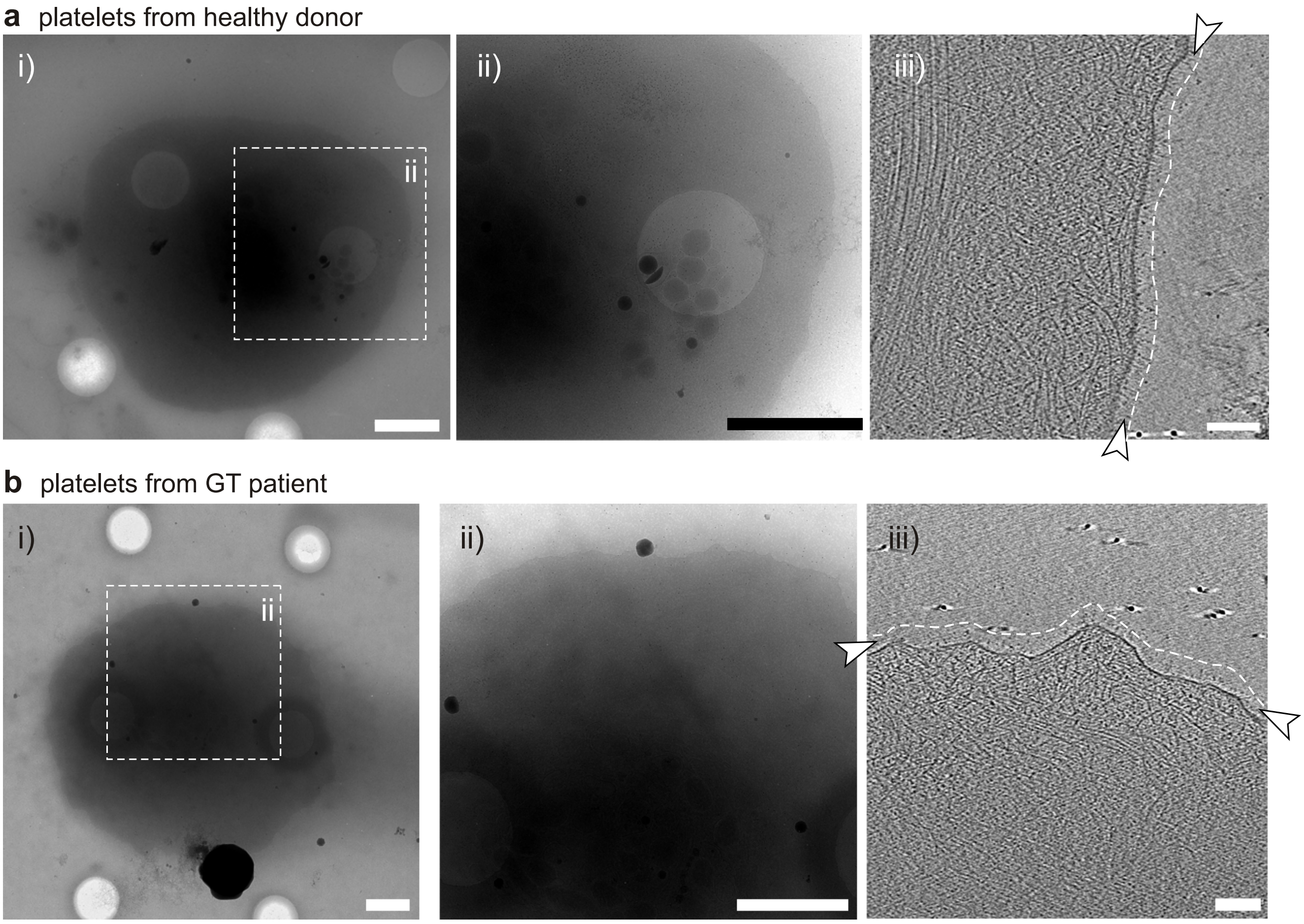

Figure S17: Cryo electron tomography of platelets from a healthy donor and a GT patient. Shown is one representative platelet from **a)** a healthy donor or **b)** the GT patient. TEM images (i and ii) were acquired at at 4’800× (i) and 11’500× (ii) magnification. Tomograms were acquired with a magnification of 42’000×, with a resulting pixel size of 0.33 nm. The XY tomographic slide shown here (iii) has a thickness of 4 nm. Arrowheads indicate the presence of membrane receptors within the membrane proximity (dashed lines) along the outline. It is possible to appreciate that the GT patient (**b**) has less membrane receptors compared to the healthy donor (**a**). Scale bars: 2 μm in i) and ii), 100 nm in iii).

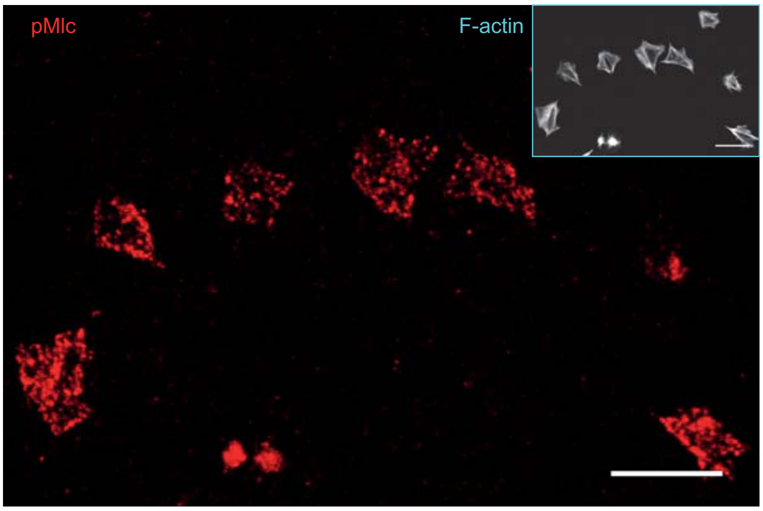

Figure S18: Phospho-myosin in healthy platelets. Immunostaining against phosphorylated myosin light chain (red). Inset: phalloidin staining. Scale bars: $10 \mu m$.

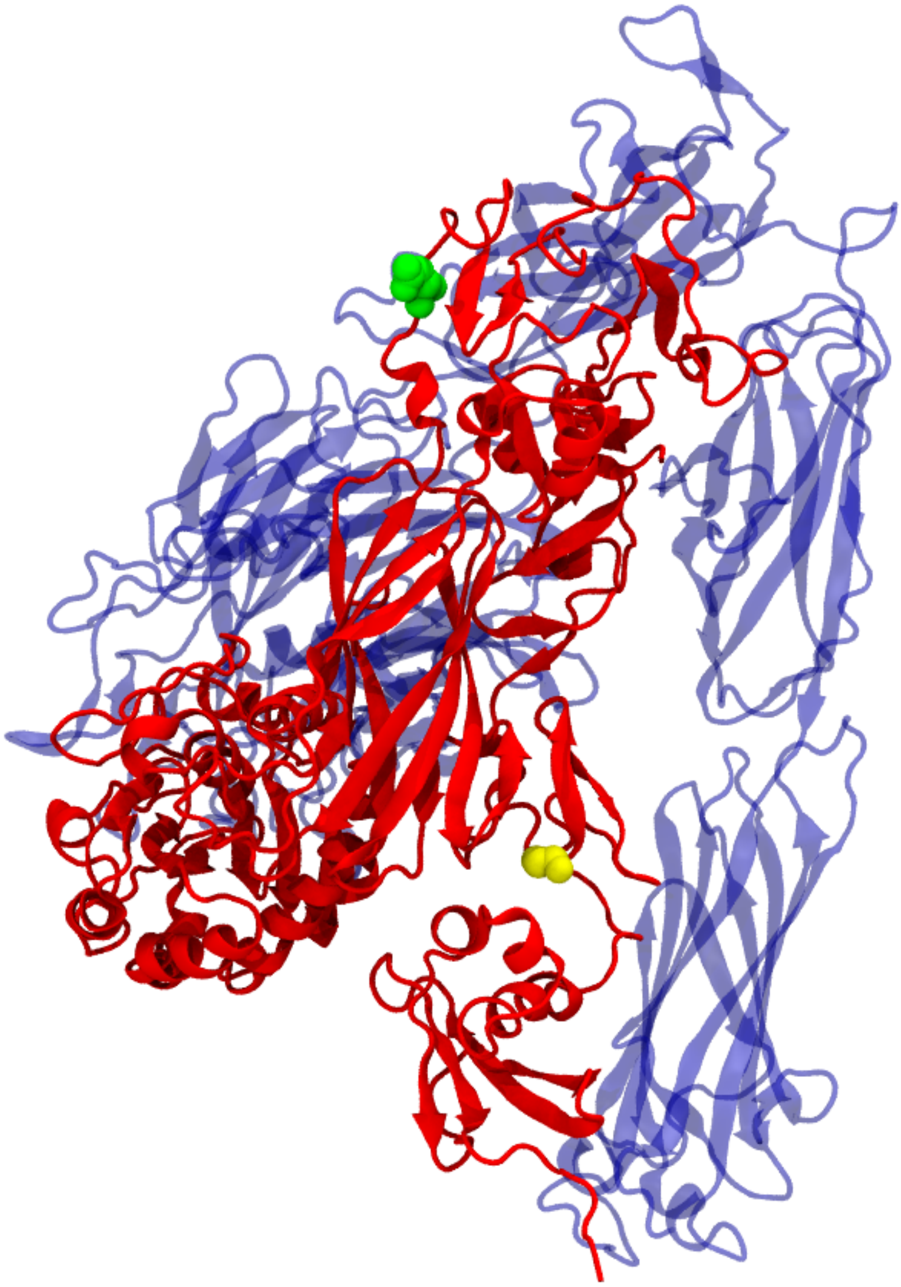

Figure S19: Location of the mutation of the GT patient. Shown are the integrin α_{IIb} (blue) and β_3 (red) subunits. The location of the missense mutations Asn470Ter (green) and Gly605Asp (yellow) in the ITGB3 gene are highlighted. PDB 3ZDY.

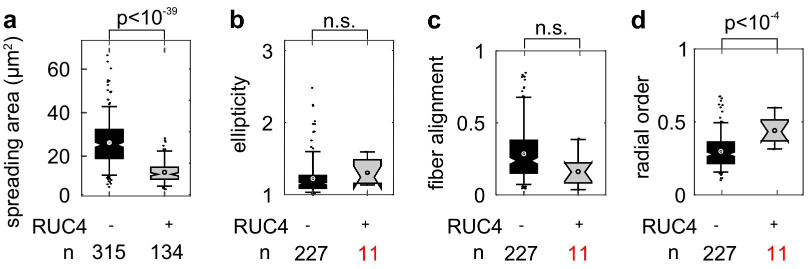

Figure S20: Spreading of platelets from a GT patient is blocked by RUC-4. Comparison between platelets of a GT patient on FG without or with 100 μM RUC-4. Shown are box plots for platelet **a)** spreading area, **b)** ellipticity, **c)** fiber alignment and **d)** radial order. RUC-4 inhibited platelet spreading and the circumferential F-actin organization, which shows that these features were mediated by integrin $\alpha_{\mathrm{IIb}}\beta_3$. The p-values in panels **b-d** should be treated with caution because only 11 GT platelets spread enough to be included in the analysis.

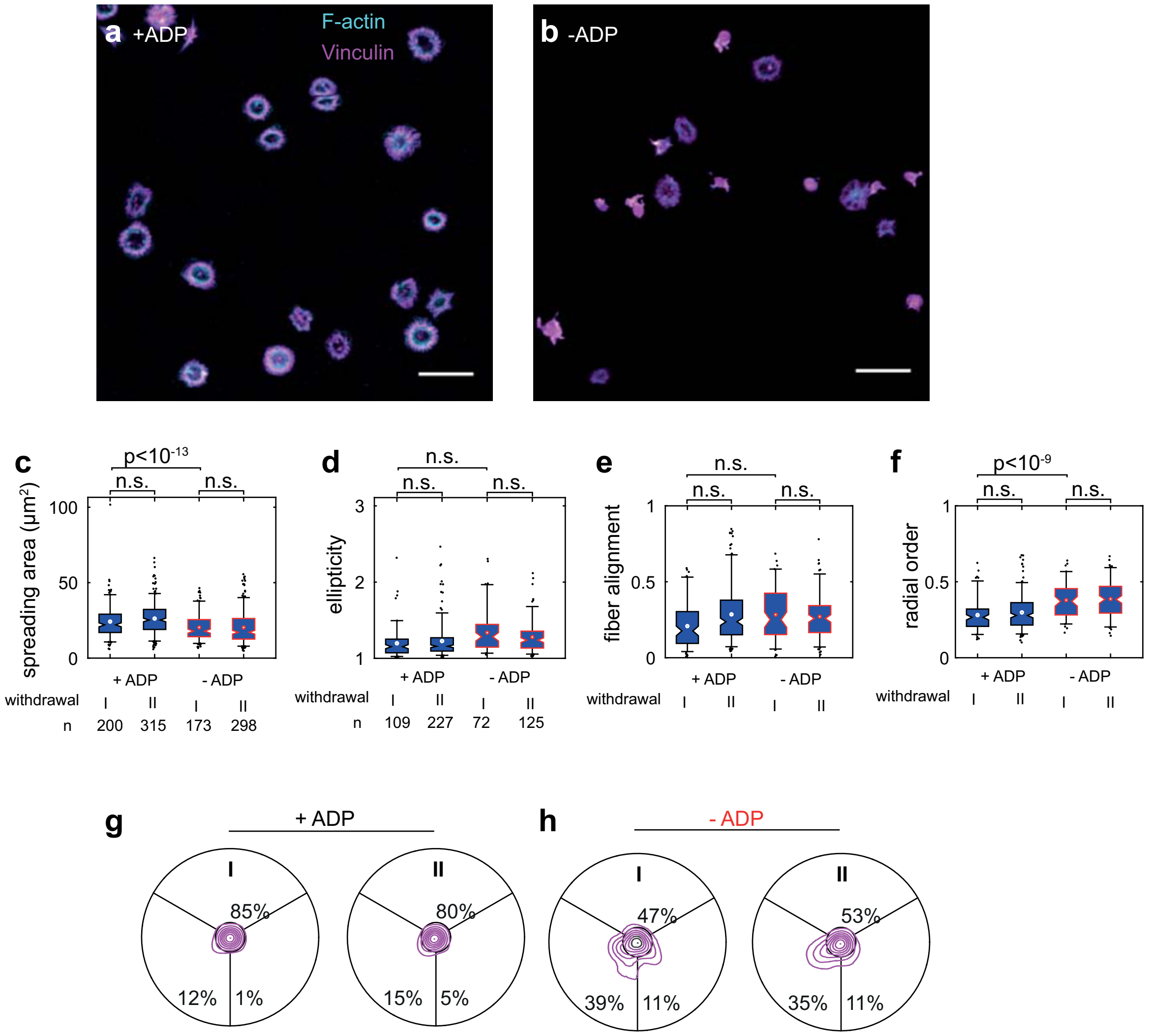

Figure S21: Morphometric analysis of GT platelets on fibrinogen with respect to ADP and repeated withdrawals. a) Confocal images of F-actin and vinculin in the presence of ADP after 60 min. **b)** Confocal images of F-actin and vinculin in the absence of ADP after 60 min. Box plots and statistical tests for **c)** spreading area, **d)** ellipticity, **e)** actin fiber alignment, **f)** actin radial order, or **g)+h)** vinculin adhesion geometry. No significant differences were detected between the two separate withdrawals I and II. In the absence of ADP, GT platelets in part failed to spread, which is reflected in a significantly smaller cell size and a less circumferential actin cytoskeleton. Scale bars: 2 μm.

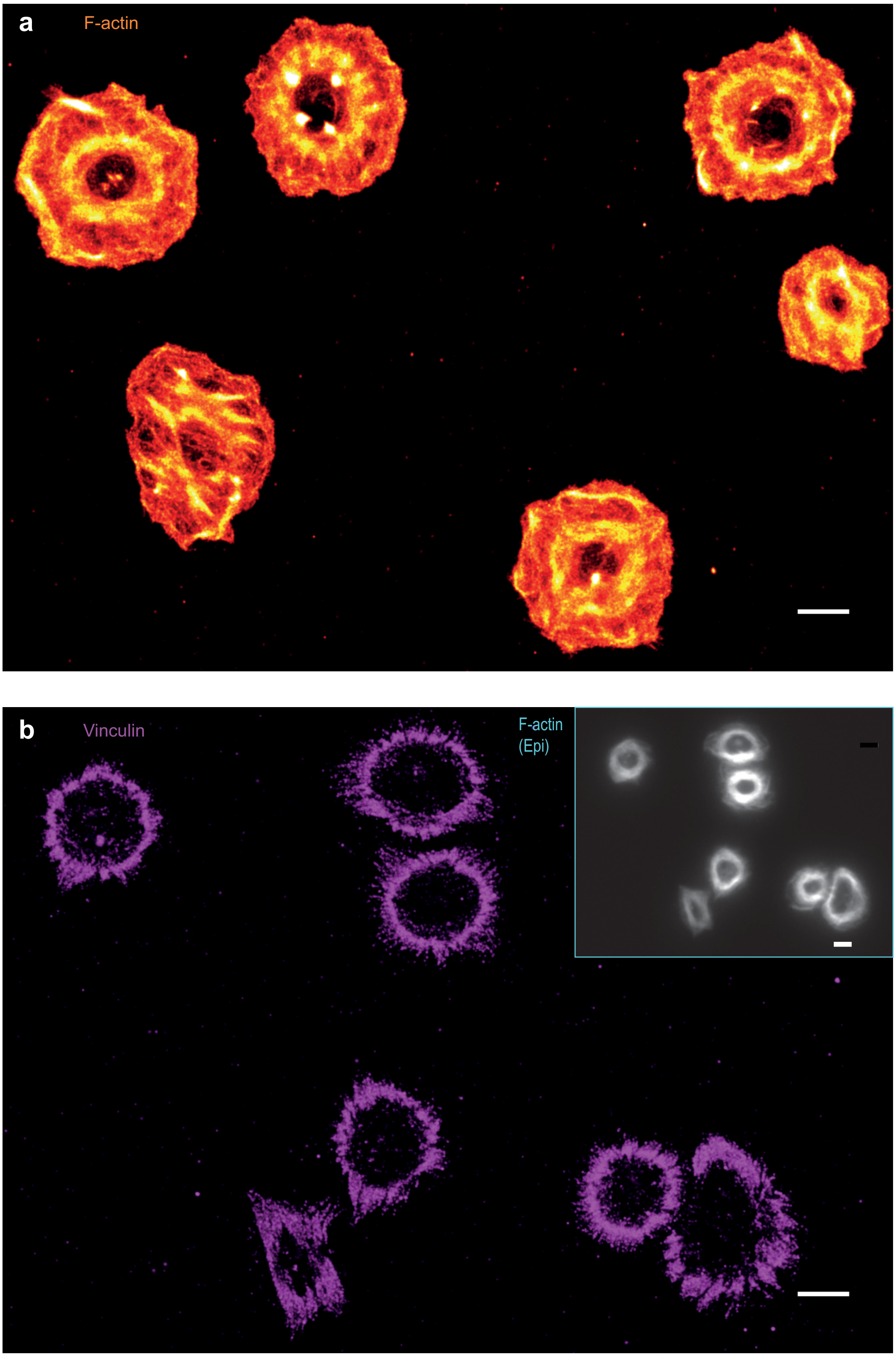

Figure S22: Superresolution (dSTORM) overview images of platelets from a patient suffering from GT: Actin and Vinculin. **a)** F-actin. **b)** Vinculin. Scale bars: 2 μm.

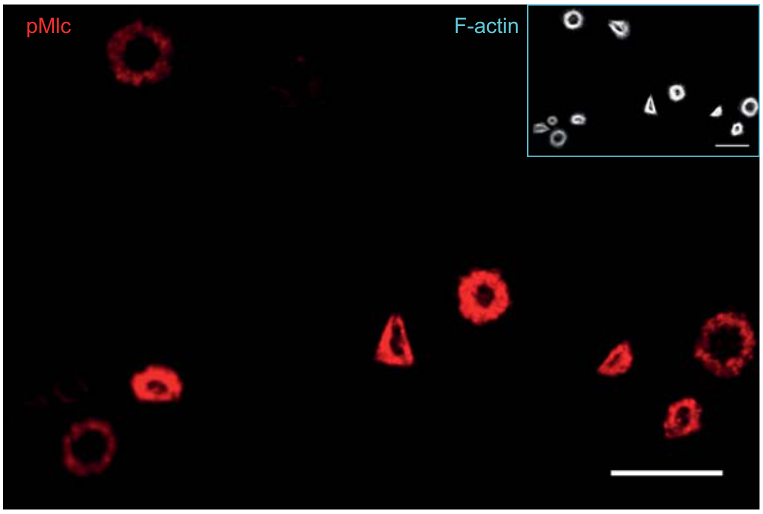

Figure S23: Phospho-myosin in GT platelets. Immunostaining against phosphorylated myosin light chain (red). Inset: phalloidin staining. Scale bars: 10 μm.

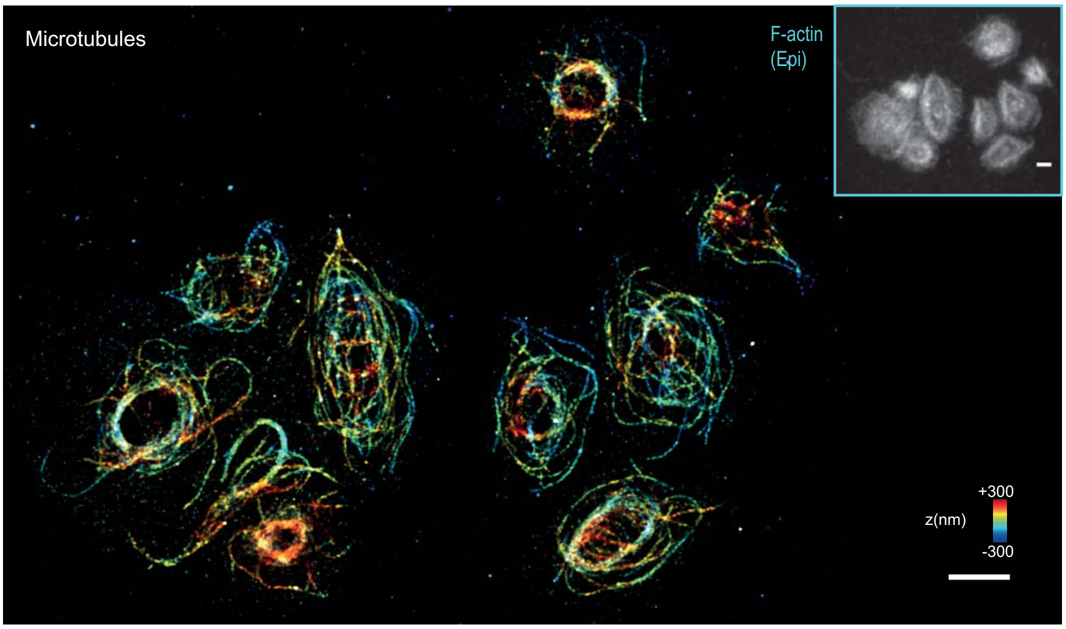

Figure S24: Superresolution (3D dSTORM) overview images of platelets from a patient suffering from GT: Microtubules. In comparison to healthy platelets (Figure S16c), microtubules of platelets from a GT patient showed less uncoiling and were more often still bundled in the marginal band, potentially as a consequence of insufficient integrin signaling/activation. Scale bars: 2 μm.

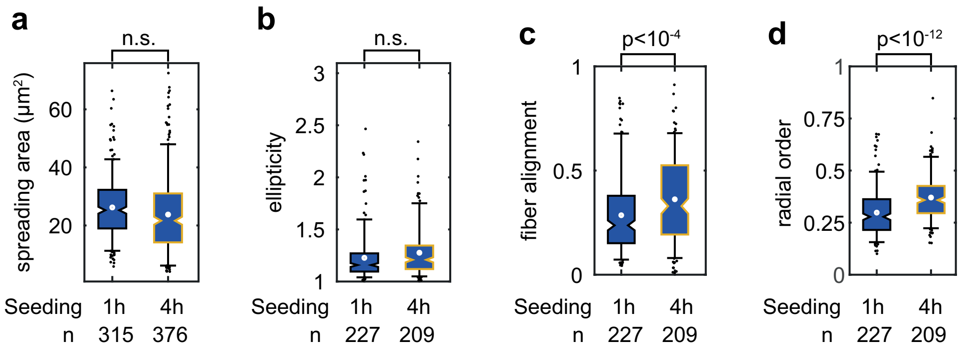

Figure S25: Influence of seeding duration on platelets from a GT patient. Platelets from a GT patient were seeded for 1 or 4 hours before fixation and staining. The influence on platelet morphology was measured in terms of **a)** spreading area, **b)** ellipticity, **c)** fiber alignment, **d)** radial order. The polarization of cells tendentially increased with prolongued seeding times in terms of their shape, actin fiber alignment and radial order, whereas their size remained constant.

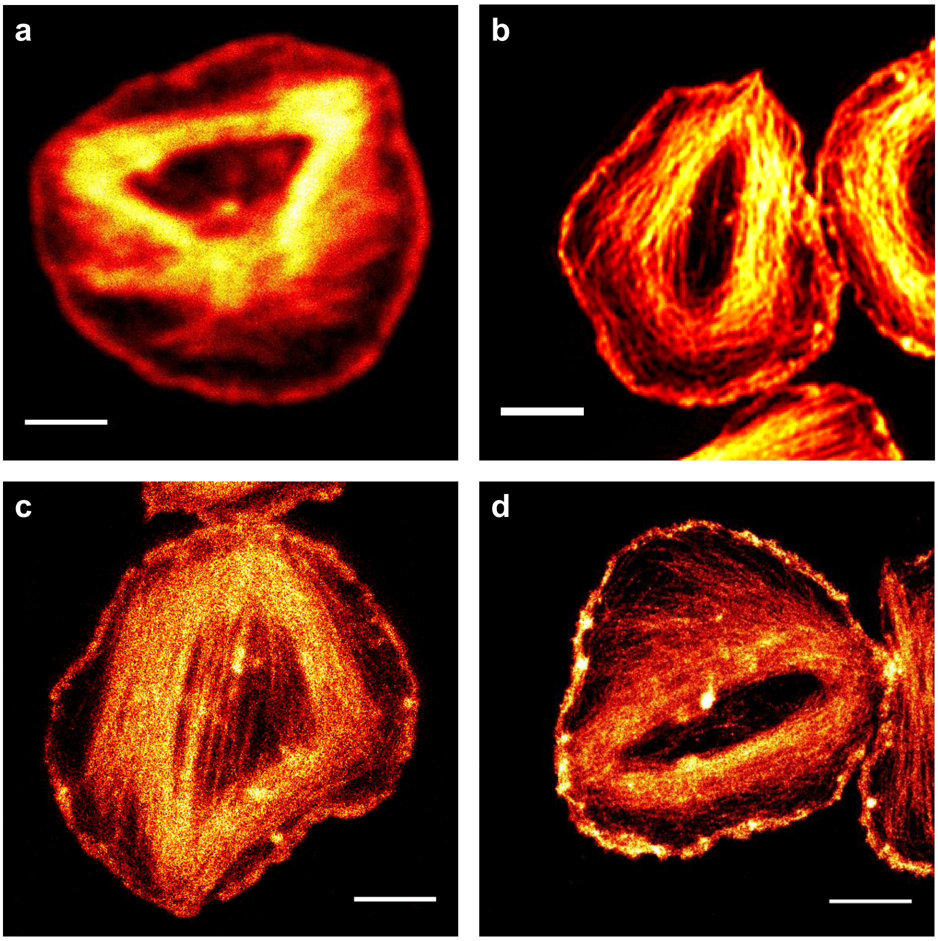

Figure S26: Comparison of confocal microscopy with superresolution microscopy methods for imaging F-actin in platelets. **a)** Confocal microscopy. Leica SP5, 63x/1.4NA oil immersion, 488 nm excitation. **b)** Structured illumination microscopy (SIM). DeltaVision OMX, 60x/1.42NA oil immersion, 488 nm excitation. **c)** Stimulated emission depletion (STED) microscopy. Leica TCS SP8 STED 3X, 100x/1.4NA oil immersion, 488 nm excitation, 592 nm depletion. **d)** Direct stochastic optical reconstruction microscopy (dSTORM). Home-built microscope, 63x/1.49NA oil immersion, 640 nm excitation. For a+b+c, F-actin was stained by phalloidin-AF488. For d, phalloidin-AF647 was used. While the increase in resolution is obvious in the dendritic actin network in lamellipodia, the gain of useful information in the bundled regions was limited. The main characteristics, i.e. the organization of strong actin bundles, were sufficiently resolved in confocal images. Scale bars: 2 μm.

2 Additional experimental methods

2.1 Buffer composition

Tyrode's buffer (TB)
TB contained 134 mM NaCl, 12 mM $NaHCO_3$, 2.9 mM KCl, 0.34 mM Na_2HPO_4, 1 mM $MgCl_2$, 10 mM Hepes, pH 7.4.

Cytoskeletal buffer (CB)
CB contained 10 mM MES, 150 mM NaCl, 5 mM EGTA, 5 mM glucose, 5 mM $MgCl_2$, pH 6.1.

dSTORM imaging buffer
The imaging buffer for dSTORM contained 200 mM Tris, pH 8.2, 4% (w/v) glucose, 1 mg ml^{-1} glucose oxidase, 0.2 mg ml^{-1} catalase, 20 mM TCEP, 2.5% glycerol and 35 mM cysteamine. The respective reagents were purchased from Sigma-Aldrich: glucose oxidase type VII (G2133); catalase from bovine liver (C4706); tris(2-carboxyethyl)phosphine (TCEP; 646547); cysteamine (30070).

2.2 Blood collection and platelet isolation

Whole blood from healthy volunteers or from the GT patient was collected in ACD tubes at the University Hospital Zurich. Purification of platelets was performed not later than four hours after blood withdrawal, if not mentioned otherwise. 6 mL blood was gently transferred into 15 mL Falcon tubes and centrifuged at 180 g for 15 minutes at RT to obtain platelet-rich plasma (PRP). 1 mL of PRP was transferred into another Falcon tube without disturbing the buffy coat layer and spun at 900 g for 5 min. The platelet-poor plasma (PPP) was carefully pipetted away and the platelet pellet was gently resuspended in 400 μL Tyrode's buffer at 37°C.

2.3 Preparation of poly-acrylamide (PAA) hydrogel substrates

Air-plasma treated glass coverslips (50mm diameter, #1.5) were incubated with 3-amino propyl triethoxy silane (APTES, A3648) at RT for 3 min, washed throroughly with water, incubated for 30 min with 0.5% (v/v) glutaraldehyde (GA, G7651) in PBS, washed again, and blown dry. 10 ml pre-gel solutions were prepared in different ratios of acrylamide and bis-acrylamide according to Tse and Engler [1], degassed for 30 minutes, and mixed with 100 μL of 10% (w/v) ammonium persulfate (APS, 215589) and 20 μL of tetramethylethylenediamine (TEMED, T7024) to initiate gelation. 3.9 μL of the activated gel solution were immediately pipetted onto an activated coverslip and a second glass coverslip (10mm diameter) was carefully placed on top. This sandwich was let to polymerize for 30 minutes and then the top coverslip was detached by a scalpel and a fine forceps. To covalently attach fibrinogen to the hydrogel surface, gels were covered with 20 μL of freshly dissolved NHS-diazirine (1 mg/mL in HEPES pH 8.2; 26167, Thermo Fisher, USA) and illuminated with UV (365nm, 0.125 W/cm^2) for 2 minutes. After four quick repetitions of this step, samples were washed in HEPES, the buffer carefully removed and 20 μL FG solution (100 μg/mL in HEPES pH 8.2) were added onto the gels for 3 hours. Gels were then washed three times with HEPES and then equilibrated in Tyrode's buffer for at least one hour. Seeding of platelets and fluorescence stainings were performed as with the normal coverslips but without mounting.

2.4 Quantification of FG surface density on PAA gel substrates

To exclude that the platelet response on hydrogels was caused by different FG surface densities, control gels of all stiffnesses were functionalized with fluorescently labeled FG following the above protocol. Confocal images showed that the staining was limited to the gel surface and that it was homogenous. For quantification of the FG amount on gels, images were taken using the same acquisition settings.

The average intensity of the FG coating varied by 20% throughout individual gels and by up to 50% between gels. No apparent trend was observed when comparing the intensities from gels with different stiffness (Supplementary Fig. S15, and differences between gels were statistically not significant.

2.5 Effect of washing steps before fixation

In our standard protocol, we washed substrates with adherent platelets only once after incubation before extraction and fixation steps. In additional experiments, we noticed that more extensive washing (four times; data not shown) removed many of the non-spread platelets observed, for example at high concentrations of integrin $\alpha_{IIb}\beta_3$ inhibitors (cf. Figure 2c for FG and FN). This explains the apparent inconsistency with previous studies that had used extensive washing and had shown that Eptifibatide or RUC-4 suppressed not only platelet spreading but also adhesion to FG or FN. The results of our morphological analysis, however, are not affected because we only analyzed platelets above a threshold spreading area of 20 μm^2 which excluded non-spread platelets from the analysis. Important to note, the gentle washing did not account for the finding that GT platelets attached to FG-coated coverslips because the spreading and attachment of GT platelets was blocked by RUC-4 (Supplementary Fig. S20).

2.6 Platelet seeding, sample preparation and fixation

Coated coverslips were placed in 12-well plates. Under standard conditions, 25 μL platelet solution was added to 500 μL pre-warmed TB containing 1 mM Ca^{2+} and 5 μM ADP and seeded on the coated coverslips at 37°C for 1 h. For titration experiments with Eptifibatide or RUC-4, the inhibitors were added to the buffer before the addition of platelets. After incubation, platelets were rinsed once with TB, detergent extracted with 0.25% (v/v) Triton X-100 and 3% (w/v) formaldehyde (FA) in cytoskeleton buffer for 90 seconds, and subsequently fixed with 3% (w/v) FA in CB for 15 minutes at RT.

For fixation of microtubules, 0.3% (w/v) and 2% (w/v) glutaraldehyde (GA) were used instead of FA during extraction and fixation, respectively, and samples were subsequently washed once and quenched with 0.1% (w/v) NaBH4 in PBS for 7 minutes. Samples for SEM were rinsed once with TB and detergent extracted with 0.75% (v/v) Triton X-100 in PHEM-buffer (60 mM Pipes, 25 mM Hepes, 10 mM EGTA, 2 mM $MgCl_2$, pH 7.4) for two minutes, then fixed with 1% (w/v) GA in PHEM for 10 minutes at RT.

2.7 Fluorescence stainings

For (immuno)fluorescence, samples were permeabilized with 0.1% (v/v) Triton X-100 and 0.5% (w/v) BSA in PBS for 10 minutes and then blocked for additional 10 minutes with 3% (w/v) BSA in PBS at RT.

For confocal microscopy, primary antibodies were diluted 1:80 in PBS containing 3% (w/v) BSA and incubated for one hour at RT, followed by three washes with PBS. Secondary antibodies (1:80) and phalloidin against F-actin (1:50) were incubated for another hour at RT, followed by three washes with PBS. Typically, phalloidin was in the 488 channel and the antibody in the 555 channel. Samples were mounted by ProLong Gold on 24 x 50 mm glass coverslips, let polymerize for 1 day at RT, and stored up to 4 weeks at 4°C.

For dSTORM, this procedure was modified as follows. Secondary antibodies were custom-labeled by NHS-reactive AF647 or CF680 yielding 1-2 dyes per antibody on average. Primary and secondary antibody concentrations were elevated (1:40), labeled samples were post-fixed with 3% (w/v) FA in PBS for 10 min, then washed and stored up to three days at 4°C in PBS. For d-STORM imaging of F-actin, phalloidin-AF647 (1:20) was incubated for 30 min, followed by three quick washes in PBS

and imaged immediately afterwards. Alternatively, phalloidin-AF488 was used as an epifluorescent co-stain. For dual-color dSTORM, AF647 and CF680 labels were used.

2.8 Stimulated emission depletion (STED) microscopy

Stimulated emission depletion (STED) microscopy (Supplementary Fig. S26c) was performed at the microscopy facility ScopeM (ETH Zurich) on a Leica TCS SP8 STED 3X instrument. The sample was stained with phalloidin-Alexa Fluor 488. A 100x/1.4NA oil immersion objective was used in combination with 488 nm excitation and 592 nm depletion. APD detection was gated 0.7 . . . 6.0 ns.

2.9 Structured illumination microscopy (SIM)

SIM (Supplementary Fig. S26b) was performed at the microscopy facility ScopeM (ETH Zurich) on a DeltaVision OMX (GE Healthcare) instrument. The sample was stained with phalloidin-Alexa Fluor 488. A 60x/1.42NA oil immersion was used in combination with 488 nm excitation. A z-stack in the range $\pm$ 5 μm was recorded and deconvolved to yield the shown image.

2.10 Scanning electron microscopy (SEM)

For SEM, GA-fixed samples were further fixed with osmium tetroxide (OsO4, 0.5% (w/v) in ddH2O) for one hour, followed by dehydration in a graded series of ethanol (from 50% to 100% in ddH$_2$O). Subsequently, the samples were dried over the critical point of CO_2 (Tc: 31°C, Pc: 73.8 bar) using a critical-point dryer (Tousimis CDP 931, USA). After sputter coating with 5 nm platinum (MED010, Balzers, Liechtenstein) images were recorded with a Zeiss Leo-1530 scanning electron microscope at 5 kV acceleration voltage detecting secondary electron signals.

2.11 Cryo electron tomography (cryo-ET)

For cryo-ET (Supplementary Fig. S17), glow discharged silicon-coated gold grids (R 1/4, Quantifoil, Germany) were placed on a drop of fibrinogen (50 μg mL^{-1}; 341576, Merck) for one hour at room temperature. Grids were washed in PBS (without Ca^{2+} and Mg^{2+}). 5 μL of platelets were added to 500 μL of warm TB with 1 mM Ca^{2+} and 5 μM ADP. And were seeded on the grids and incubated for 40 min at 37 °C. The grids were then plunge-frozen in liquid ethane.

Data acquisition was performed at on FEI Titan Krios, 300 kV transmission electron microscope equipped with a Gatan Quantum Energy Filter and a K2 summit direct electron detection camera. The tilt series were acquired from $-60°$ to $+60°$ with 2° tilt increments, using serial EM software, at 8 μm defocus and a magnification of 42,000$\times$, resulting in a pixel size of 0.33 nm. The cumulative electron dose was about 60 e^{-}Å^{-2}.

3 Image analysis

Image analysis was done in a largely automated fashion with custom-written MATLAB scripts (The MathWorks), if not mentioned otherwise. All scripts and procedures were optimized with respect to yielding robust results which were not sensitive to moderate changes in the choice of threshold parameters. Input images were 16 bit, 2048x2048 pixel large confocal scans with an effective pixel size in the image plane of 60.1 $\times$ 60.1 nm.

As a first step before all subsequent morphological operations, the raw fluorescent actin image $I_{\mathrm{actin}}^{\mathrm{raw}}$ was converted to real numbers, preprocessed by a 3x3 median filter, and normalized to $[0, 1]$ by

$$I_{\text{actin}} = \frac{I_{\text{actin}}^{\text{raw}} - \min\left(I_{\text{actin}}^{\text{raw}}\right)}{\max\left(I_{\text{actin}}^{\text{raw}}\right) - \min\left(I_{\text{actin}}^{\text{raw}}\right)} \tag{1}$$

The logical conception of our image analysis is summarized in Supplementary Fig. S2. The analysis of cell outlines (see section 3.1), actin fibers (see section 3.2.1) and their orientation (see section 3.2.2) was performed on the full images. Further image analysis steps for determining single cell characteristics were restricted to an ROI containing a single cell.

3.1 Cell outline

For determining cell outlines (Supplementary Fig. S2, step 1 → 2), a threshold was defined based on the background intensity as follows. The image I_{actin} was normalized to the 99.5th percentile of all image pixel values; this step ensures that the intensity scaling of the image is not dominated by small very bright aggregates or cells. A histogram was then made of all image pixel values in the range $(0, 0.1)$ with 0.005 bin width. The intensity value of the bin which had the maximum counts was defined as the dominant background level bg. The standard deviation of the background peak sd_{bg} was calculated from all values between zero and $2bg$. The image was then binarized according to the threshold $th_{\text{outline}} = bg + f \cdot sd_{\text{bg}}$. The multiplicative factor f was typically chosen around ~ 10; this choice ensured that the position of the outline was neither too tight (cutting off dim regions of the cellular periphery, e.g. for $f > 30$) nor too loose (incorporating background into the outline, e.g. for $f < 0.05$). Within this range, the outline determination was quite robust against changes in the choice of this factor and the contrast of individual images.

The binarized outline mask was postprocessed as follows. An image opening operation was performed with a disk-shaped structuring element with radius 5; this step removes thin bridges between touching objects. Objects smaller than $A_{\text{obj}}^{\text{min}} = 1000$ pixels $(= 3.6\mu m^2)$ were removed, and a morphological image reconstruction based on the remaining seeds and the original mask was performed to undo the shrinking of the first step. Holes which were smaller than $A_{\text{hole}}^{\text{max}} = 1000$ pixels were filled; this step was especially necessarry to fill in the often dark central part of platelets with a ring-shaped or triangular actin cytoskeleton. Object outlines were smoothened by processing the mask with a 7x7 median filter, which does not displace the position of a straight outline. Finally, once more objects that were smaller than $A_{\text{obj}}^{\text{min}}$ were removed.

These automatically created masks were visually checked and manually corrected for errors or touching cells in a custom-written LabView GUI. Objects in the final resulting masks M_{outline} were directly used to calculate single cell spreading area, ellipticity (defined as the ratio between major and minor axis of an equivalent fitting ellipse), and circularity. They also were used to restrict the further analysis of the actin cytoskeleton and of other stainings to the single cell of interest.

3.2 Actin cytoskeleton

3.2.1 Actin fibers

For restricting the orientation analysis to fibers (Supplementary Fig. S2, step 1 → 3), features in raw actin intensity images were enhanced by graded unsharp masking as follows. Smoothened versions of the preprocessed actin intensity image were created by filtering with Gaussian masks of different sigma (3, 5, 7, and 9 pixel). Each smoothened image was subtracted from the original image, and all difference images were summed up. A 3x3 median filter was applied to reduce salt-n-pepper noise followed by a single 3x3 unsharp masking operation. Then Gaussian smoothing with 2 pixel sigma was applied followed once more by a single 3x3 unsharp masking operation. The resulting image was normalized to 1 and thresholded at a user defined level th_{fib} of typically 0.08. The final result was a binary mask M_{fibers} that contained thin or small objects with sufficient contrast which are, in the case here, predominantly actin fibers.

3.2.2 Cytoskeletal orientation

For determining the local orientation in preprocessed actin images (Supplementary Fig. S2, step 1 $\rightarrow$ 4), a filtering method was used that takes the advantage of detecting the 2nd order gradient (curvature) in actin intensity images. The basic elements are 5x5 Sobel kernels in the x direction S_x and in the y direction $S_y = S_x'$ with

$$S_x = \begin{bmatrix} 1 & 2 & 0 & -2 & -1 \\ 2 & 4 & 0 & -4 & -2 \\ 3 & 6 & 0 & -6 & -3 \\ 2 & 4 & 0 & -4 & -2 \\ 1 & 2 & 0 & -2 & -1 \end{bmatrix} \tag{2}$$

and S_x' being the transposition of S_x. Based on these, three filter operations $S_{xx} = S_x \cdot S_x$, $S_{yy} = S_y \cdot S_y$, and $S_{xy} = S_x \cdot S_y$ were constructed where the dot product here represents sequential application of the filter kernels on the image. Processing of the actin image with S_{xx}, S_{yy}, and S_{xy} resulted in three filtered images, I_{xx}, I_{yy}, and I_{xy}, respectively, which were then combined into curvature images $I_1 = (I_{xx} - I_{yy})$ and $I_2 = -2I_{xy}$. These images were each multiplied pixelwise with the preprocessed actin intensity image, smoothened by a Gaussian blur with 4 pixel sigma, and normalized pixelwise with a smoothened intensity image of the same blurring degree, resulting in smoothened curvature images I_1^* and I_2^*, respectively. The local orientation ϕ was then computed for each pixel in the whole image I_ϕ by taking the quadrant-sensitive arcus tangens:

$$I_\phi = 0.5 \tan^{-1}\left(\frac{I_2^*}{I_1^*}\right). \tag{3}$$

The values of the resulting actin orientation image I_ϕ lay between $-\pi/2$ and $+\pi/2$.

3.2.3 Radial orientation

To assess whether actin fibers are oriented circumferentially or radially, the center of mass of the mask of a single cell was taken as the center of the cell, (x_0, y_0). Each pixel within the cell outline was assigned an angle ψ according to its position relative to this central pixel (Supplementary Fig. S2, step 2 $\rightarrow$ 6):

$$\psi = \tan^{-1}\left(\frac{y_i - y_0}{x_i - x_0}\right) \tag{4}$$

The values of the resulting image I_ψ lay between $-\pi/2$ and $+\pi/2$. The local relative orientation ϕ of the actin fibers (Supplementary Fig. S2, image 8) with respect to the radial reference direction ψ (Supplementary Fig. S2, image 7) was quantified by a radial order parameter S_{radial} defined as

$$I_S^{\text{radial}} = 0.5 \cos\left(2\left(I_\phi - I_\psi\right)\right) + 0.5 \tag{5}$$

(Supplementary Fig. S2, step $6+7 \rightarrow 8$). The values of the resulting radial actin orientation image lay between 0 and +1, meaning that an actin fiber is locally perpendicular to or aligned with the radial direction, respectively.

3.2.4 Calculation of single cell order parameters

To quantify the degree of self-alignment of the actin cytoskeleton in single platelets, which can also be understood as a measure of cytoskeletal polarization, a single cell actin alignment parameter S^{self} was calculated as follows. Four images were used: the outline mask of the single cell M_{outline} (Supplementary Fig. S2, image 2), the corresponding mask for actin fibers M_{fibers} (Fig. S2, image 3), its actin orientation image I_ϕ (Supplementary Fig. S2, image 4), and the preprocessed actin intensity image I_{actin} (Supplementary Fig. S2, image 1). First, the intensity-weighted average of the orientation within only the fibrillar regions of this cell (Supplementary Fig. S2, image 8) was calculated by

$$\phi_0 = \arg\left[\frac{\sum \exp\left(iI_\phi\right) \cdot M_{\mathrm{outline}} \cdot M_{\mathrm{fibers}} \cdot I_{\mathrm{actin}}}{\sum M_{\mathrm{outline}} \cdot M_{\mathrm{fibers}} \cdot I_{\mathrm{actin}}}\right] \tag{6}$$

Here, all matrix multiplications are element-wise, the sum runs over all image pixels, and $\arg(z) = \tan^{-1}\left(\Im(z)/\Re(z)\right)$. The orientation of fibers relative to this average orientation ϕ_0 then was calulated as $I_\phi^* = \arg\left[\exp(iI_\phi) - \exp(i\phi_0)\right]$. From this, the fiber alignment parameter was calculated in an analogue way, viz. weighted by the actin intensity:

$$S^{\mathrm{self}} = \left[\frac{\sum \cos\left(2I_\phi^*\right) \cdot M_{\mathrm{outline}} \cdot M_{\mathrm{fibers}} \cdot I_{\mathrm{actin}}}{\sum M_{\mathrm{outline}} \cdot M_{\mathrm{fibers}} \cdot I_{\mathrm{actin}}}\right] \tag{7}$$

The fiber alignment parameter lies between 0 (isotropic, or disorganized) and +1 (completely anisotropic, or polarized, actin cytoskeleton).

As an additional quantification of the overall actin organization, an average radial actin order parameter, weighted also by the actin intensity, was calculated from the radial actin image (Supplementary Fig. S2, image 10) as follows:

$$S^{\mathrm{radial}} = \frac{\sum I_S^{\mathrm{radial}} \cdot M_{\mathrm{outline}} \cdot M_{\mathrm{fibers}} \cdot I_{\mathrm{actin}}}{\sum M_{\mathrm{outline}} \cdot M_{\mathrm{fibers}} \cdot I_{\mathrm{actin}}} \tag{8}$$

This parameter lies between 0 (ring-shaped) and +1 (star-shaped cytoskeletal morphology).

3.3 Vinculin

3.3.1 Circumferential profile of adhesions

To quantify how the formed cell-substrate adhesions are distributed around a single cell, the cell area was devided into 20 equal angular sectors originating at the cell center (x_0, y_0) (Supplementary Fig. S2, image 5). Pixel values from the original vinculin image (Fig. S2, image 9) were averaged for each sector, and these values were normalized to their sum and plotted according to their circumferential position, resulting in a circumferential profile (Supplementary Fig. S2, image 10, data points). These data were then further fitted to classify the adhesion morphology into parallel, triangular, or other shapes (see next section).

3.3.2 Classification of adhesion morphology

The vinculin stainings correlated with the end of actin fiber bundles. They thus were used further to classify the adhesion morphology in an attempt to quantify whether the contractile actin cytoskeleton was predominantly polarized in a dipole fashion (=parallel bundles with adhesions at two opposite sides of the cell), in a triangular fashion (=bundles arranged between adhesions at three sites of the cell), or rather unpolarized (=an actin ring connecting with many adhesions all-around, or star-shaped

actin bundles connected to similarly distributed adhesions). To this end, the circumferential intensity profile of vinculin was obtained (see section 3.3.1) and fitted to a fourier series up to fourth order:

$$Y(\alpha) = 1 + \sum_{k=1}^{4} a_k \cos(k\alpha) + b_k \sin(k\alpha) \tag{9}$$

Here, the kth Fourier component has weight $w_k = \sqrt{a_k^2 + b_k^2}$; the zeroth component was set to one due to the preceding normalization of the intensity profile. Supplementary Fig. S2, item 13, displays an example for the fit (red line) and the individual contributions from the 2nd, 3rd and 4th Fourier components (gray lines). In this particular example, the fitted weights were $w_2 = 0.23$, $w_3 = 0.96$, and $w_4 = 0.25$. The dominance of the third Fourier component over the other two reflects the triangular adhesion morphology of the analyzed cell.

3.3.3 Overview plot of adhesion morphology

To visually display the most important characteristics of the adhesion morphology of single platelets, we created a custom overview plot (Supplementary Fig. S3). In the basic version, each platelet is represented as a point in a circular region.

1. The direction at which the point is placed is determined by the relative weights of the Fourier components as derived from the vinculin staining (see section 3.3.2). The positions are derived from $x_i = \sqrt{3}/2\,(w_3 - w_2)$ and $y_i = w_4 - 0.5\,(w_3 + w_2)$ and then normalized to yield $\tilde{x}_i = x_i/(x_i^2 + y_i^2)^{0.5}$ and $\tilde{y}_i = y_i/(x_i^2 + y_i^2)^{0.5}$, respectively.
2. The radial position of the point is determined by the distinctness of the adhesion arrangment. From the fit of the vinculin circumferential intensity, the amplitude was determined by the maximum value of the function

$$Y(\alpha) = \sum_{k=2}^{4} a_k \cos(k\alpha) + b_k \sin(k\alpha) \tag{10}$$

   on the interval $[-\pi, \pi]$. This is essentially the same as the amplitude of the original fit but neglects the first component that only accounts for unequal brightness from one to the other side. To correct for a baseline in washy stainings, 0.25 was subtracted which is approximately the minimum observed amplitude of all cells. The absolute value of the resulting parameter was then used to radially scale the $(\tilde{x}, \tilde{y})$ positions.

The resulting graph can be understood in the following way: dipolar cell morphologies are found in the lower left third, triangular ones in the lower right third, quadratic ones in the upper third. All others are found in the in the middle. Supplementary Fig. S3a+b shows some examples of how the points in the overview plot refer to the underlying vinculin stainings.

3.4 Summary morphology plots of platelet populations

3.4.1 Correspondence between vinculin and actin morphological parameters

The establishment of (vinculin cointaining) focal adhesions and the organization of the actin cytoskeleton are tightly linked. Both together determine the functional contractile morphology.

Supplementary Fig. S3c+d show that dipolar adhesion morphologies are accompanied by a highly aligned actin cytoskeleton, whereas the alignment decreases for triangular cells and is very low for cells in the central middle region. Supplementary Fig. S3e shows that the actin cytoskeleton is preferentially radially organized for dipolar adhesion morphologies (left sector), circumferential for triangular

morphologies (right sector), and can be either (stellar) or (ring-shaped) for cells with a relatively symmetric arrangement of focal adhesions (middle).

3.4.2 Derived density plots

With increasing number of cells the above representation (Supplementary Fig. S4a) becomes ever more difficult to read. We thus use density plots which are proportional to the number of cells with similar morphology and give an intuitive impression of the distribution within a platelet population.

1. For the density plot, the $(\tilde{x}, \tilde{y})$ positions of all cells were binned into 0.02 wide bins, averaged by a Gaussians with radius 0.15, and normalized to the maximum value. The resulting density was constructed by height lines and overlaid with a grayscale color-code (Supplementary Fig. S4b).

   Healthy platelets predominantly adopted a dipolar morphology, coexisting with a minor triangular population.

2. For a averaged map of the actin alignment, cells were binned into 0.05 wide bins according to their alignment parameter. For each of these subgroups, a density plot was constructed and averaged as described above. Finally, a weighted average sum of the alignment parameter was calculated pixelwise using the smoothened binned density plots as weights, and normalized to the pixelwise sum of these density images. The resulting map was color-coded using the "parula" colormap clipped to the range $[0.2, 0.8]$, and overlayed with the contours of the density plot (Supplementary Fig. S4c).

   The filament alignment decreases from polarized cells (to the left) to triangular morphologies (to the right) to isotropic cytoskeletal arrangements (in the middle).

3. An averaged map of the radial order parameter was created analogously to that for the alignment parameter. The resulting map was color-coded from blue to red, clipped to the range $[0.25, 0.75]$, and overlayed with the contours of the density plot (Supplementary Fig. S4d).

   Isotropic cells in the middle tend to have both radial as well as circumferential arrangements, triangular cells go along with a circumferential actin order, and the most pronounced dipolar cells tend more towards a radial arrangement.

3.5 Movies of live cell spreading

Confocal live cell movies were post-processed for visualization. As the fluorescence background of the coverslip increases over the time course of the movie probably due to binding of SiR actin, we subtracted it. To this end, a background region was determined by intensity thresholding the last frame in the movie at the lower of a 2-level Otsu threshold, median filtering of the binary image with a 5x5 window, dilation with a disk-shaped structuring element of radius 7, filling of holes, and invertion of the selection. Using this mask, the mean intensity of background pixels was extracted for each frame. The intensity-time-trace was subjected to median filtering with a window size of 11 and then normalized to the intensity of the first frame. A background image was obtained by avering the first four frames of the movie. This background image was scaled by the renormalized intensity factor for a respective frame and subtracted from the corresponding original image. The image stack was loaded into ImageJ and cropped to the region of interest. Contrast enhancement with 0.3% saturated pixels was applied to each slice individually. Selected slices were arranged into a montage and finally median filtered with a radius of 1.

4 Statistical analysis

The following single cell properties were subjected to further analysis: spreading area, ellipticity, filament order, and radial order. Moreover, morphology plots derived from vinculin adhesion stainings were compared by a cross-correlation.

4.1 Box plots

Box plot are used to represent the distribution of a single morphological parameter. Boxes represent upper and lower quartiles q_3 and q_1. Notches depict the median q_2 and the comparison intervals with extremes at $q_2 \pm 1.57(q_3 - q_1)/\sqrt{n}$. The mean is depicted as a small circle. Whiskers represent the 5th and 95th percentiles, and data outside of these are depicted as dots.

4.2 Multiple comparisons

When comparing the distributions of a single property between different donors or conditions, the data were first checked for normality and equal variances using the Brown-Forsythe test. As some parameters did not follow a normal distribution, we applied a non-parametric Kruskal-Wallis rank test with Scheffe post-hoc testing. As a general remark, Kruskal-Wallis with Scheffe was always less discriminative than ANOVA with Tukey-Kramer; our conservative choice thus might underestimate the effect size for some cases.

4.3 Comparison of morphology plots

The morphology density plots are a useful visual representation of the distribution of different platelet morphologies within a population. To quantitatively compare how similar different density plots are, we used a normalized cross-correlation. To this end, we binned the platelets into a 0.02 pitch grid and smoothened the distribution as described in section 3.4 with the only difference that the resulting density plot D was normalized to yield an autocorrelation of 1

$$\tilde{D} = \frac{D}{\sum (D \cdot D)} \tag{11}$$

where $\cdot$ denotes elementwise matrix multiplication and the sum runs over all bins.

The cross-correlation $C_{i,j}^{\text{morph}}$ between two density plots $\tilde{D}_i$ and $\tilde{D}_j$ was then simply obtained from elementwise multiplication and summation:

$$CC_{i,j}^{\text{morph}} = \sum \tilde{D}_i \cdot \tilde{D}_j \tag{12}$$

4.4 Construction of similarity trees

For a quantitative and visual analysis of similarity between different morphologies, dendrograms were constructed by the following procedure. As a measure for the 'distance' between two morphology density plots, we took $1 - CC_{i,j}^{\text{morph}}$. From all pairwise distances, we constructed a 'phylogenetic' tree by sequential linkage (=hierarchical ascendent classification) using the `seqlinkage` command in MATLAB with the distance method *'average'*.

4.5 Coefficient of variation (CV)

The coefficient of variation between different samples in a dataset X is in general defined by the standard deviation divided by the mean:

$$CV\left(X\right) = \frac{\sqrt{Var\left(X\right)}}{\left\langle X\right\rangle} \tag{13}$$

This formula was directly applied to the spreading area, the filament alignment, and the radial order. For the ellipticity, we divided by $\langle X\rangle - 1$ to correct for the fact that the ellipticity by definition is always greater than 1; otherwise, the CV would be underestimated. For CC^{morph}, the CV describes how large the variation of the similarity between pairs of morphology plots was, rather than how large the variation was between the plots themselves (because this is quantified by the cross-correlation itself).

4.6 Dose-response curves

For fitting of dose-response curves, concentrations (given in μM) were converted into a logarithmic scaling by taking the decadic logarithm $\log(c)$. Using these as x-values, the data were fitted by a 4-parameter logistic curve according to the Hill equation:

$$Y(x) = p_1 + \frac{p_2 - p_1}{1 + 10^{(p_3 - x)p_4}} \tag{14}$$

Here, the fitting parameters p_i have the meanings: p_1 = minimum saturation value, p_2 = maximum saturation value, p_3 = EC_{50} or IC_{50}, and p_4 = the slope of the Hill curve, with negative values for inhibition. In addition to the fitted values, the 95% confidence interval is given for each value/curve.

Supplementary References

[1] J. R. Tse and A. J. Engler. "Preparation of hydrogel substrates with tunable mechanical properties". In: *Current Protocols in Cell Biology* 47.10.16 (2010), pp. 1–16. ISSN: 19342500. DOI: 10.1002/0471143030.cb1016s47.
