# Supplementary material for: Morphometric analysis of spread platelets identifies integrin αIIbβ3-specific contractile phenotype
Source: Sci Rep. 2018 Apr 3;8:5428. doi: 10.1038/s41598-018-23684-w (PMC5882949; doi:10.1038/s41598-018-23684-w)
Supplement: Supplementary file 3 — Dataset 1 [file 41598_2018_23684_MOESM3_ESM.zip › Workflow_Morphometrics/MorphometricPlateletScreen_Userguide.pdf]

Morphometric Phenotyping

Step-by-Step Guide

This user guide and the respective MATLAB code accompany the article
S. Lickert et al., "Morphometric analysis of spread platelets identifies integrin αIIbβ3-specific contractile phenotype", Scientific Reports, 2018.
Please cite this work when using the code for your research project.
©2018 by Sebastian Lickert and Ingmar Schoen. Email: ingmarschoen@rcsi.ie

1 Requirements

This software has been developed on a Windows 10 computer with **MATLAB** 2017a.
The following MATLAB **toolboxes** are required:

- Image processing
- Curve fitting
- Statistic and machine learning
- Bioinformatics

The provided MATLAB **code** encompasses
Top-level functions (description see below):

- Define_cells.m & Define_cells.fig (GUI)
- Edit_cells.m & Edit_cells.fig (GUI)
- Process_Cells.m
- Combine_Populations.m
- Display_Morphometrics.m
- Display_Comparison.m
- Display_DoseResponse.m

Low-level functions (description see comments in code):

- angdiff2lines.m
- boxplotIngmar.m
- cellstats2mat.m
- circprofile.m
- cropim2obj.m
- getfourieramplitudes.m
- periodicsinefit.m
- plotmeans.m
- putvar.m
- pvaluetableKW.m
- radialorderimages.m
- redblue.m

In addition, you need to have **Ghostscript** installed on your machine.

The image analysis has been optimized for images with a **pixel size** of 60 nm. Adaptions to other pixel sizes might require an adaption of filtering steps, i.e. for the determination of the actin orientation.

2 Basic Folder Structure

Define_Cells

This part of the software creates an outline of each cell and detects the fibers based on the actin stain.

Edit_Cells

With this function, you can manually correct the automatically generated outline masks, delete confluent cells or aggregates and separate touching platelets.

Process_Cells

In this part the general image analysis is done. Furthermore, it is possible to combine processed datasets into larger datasets for pooling, comparison, or fitting.

Visualize_Cells

A collection of functions to visualize morphological parameters and contour plots of the adhesion site distributions, as well as to statistically compare datasets and generate dose-response curves.

3 Workflow

For an intuitive understanding the workflow is illustrated with example data, attached in the folder "Workflow_Morphometrics".

3.1 Image preparation

Process the images into single "-.tiff"-files. Use for each stack the same base name and add an identifier for each staining/channel.

Please note that you need a consistent and unique naming convention to identify the different channels. This can be "[*]Actin.tif" or "[*]ch01.tif" or something similar. We always have used the first version and have not rigorously tested other options.

Name	Date modified	Type	Size
Exp_x_01_Actin.tif	06.04.2017 10:09	TIFF image	8 628 KB
Exp_x_01_Vinculin.tif	06.04.2017 10:09	TIFF image	8 628 KB
Exp_x_02_Actin.tif	06.04.2017 10:09	TIFF image	8 628 KB
Exp_x_02_Vinculin.tif	06.04.2017 10:09	TIFF image	8 628 KB

3.2 Matlab

Open Matlab 2015b or newer.

Select the folder "Workflow_Morphometrics".

Add this folder and its subfolders to the path.

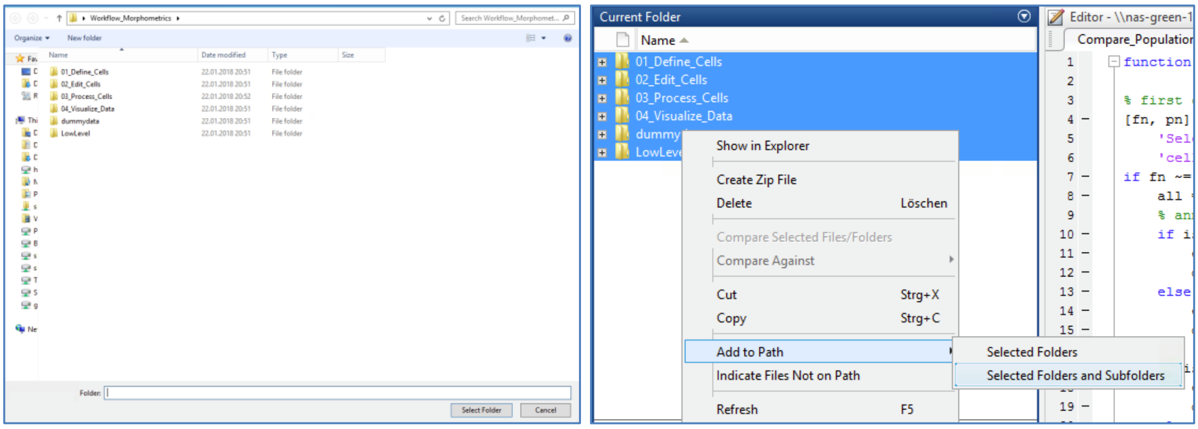

3.3 Defining cells

Open the folder "01_Define_Cells" and run the code "Define_Cells.m" that opens a GUI.

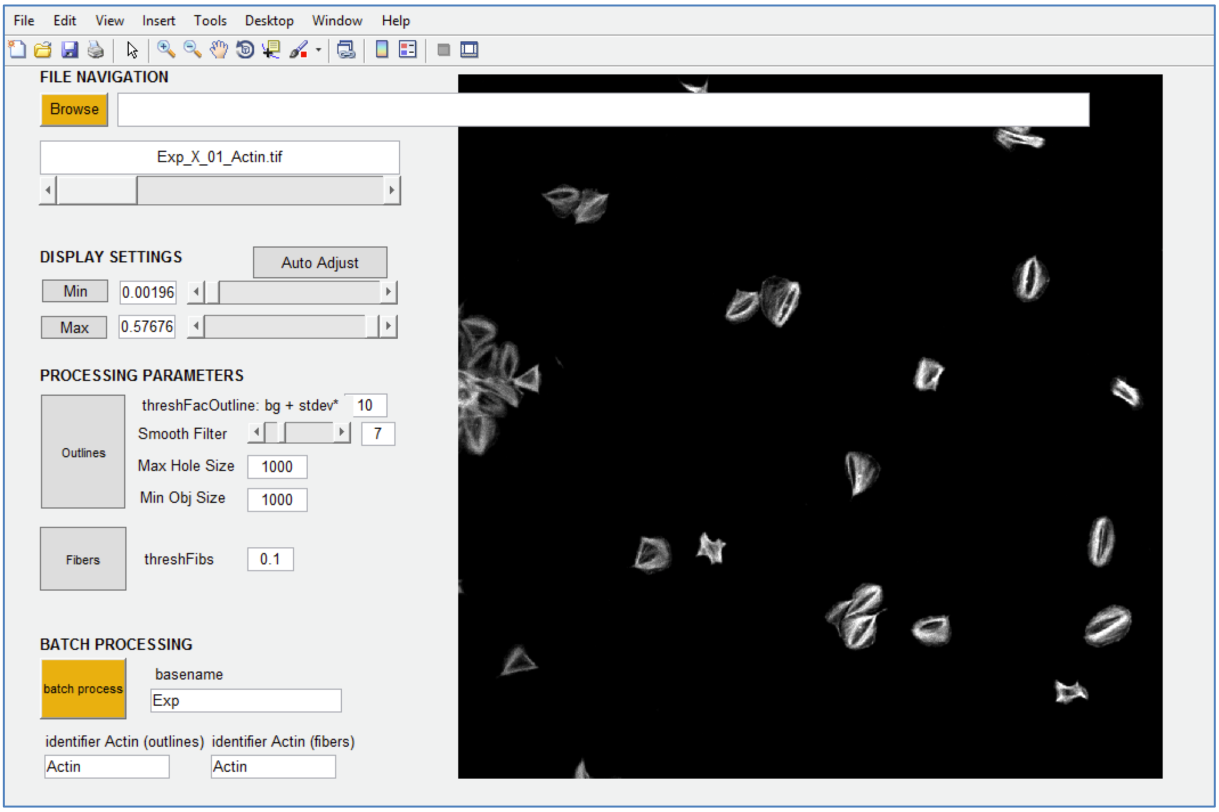

Under *FILE NAVIGATION*, click **Browse** and select the folder containing the images. Use the sliding bar beneath the current image to go through all images in the folder.

Under *DISPLAY SETTINGS*, you can **adjust** the contrast of the displayed image.

Under *PROCESSING PARAMETERS*, you can adjust and test the creation of the outline mask and of the fiber mask.

By pressing **Outlines** you can check the quality of the outline detection.
threshFacOutline: multiplicative factor that us used to determine the threshold, based on the mean and the standard deviation of the background. Default: 10. Range: 0…Inf. Decrease to enlarge objects, increase to obtain tighter outlines.
SmoothFilter: size of the median filter (in pixels) that is used to smoothen the generated outlines. Default: 7. Range: uneven integers 1,3,5,… Enlarge to make outlines smoother.
MaxHoleSize: maximum size (in pixels) of void regions that are filled. Voids larger than this value will remain. Default 1000. Depends on effective pixel size. In our experience, a value corresponding to 3.5 μm^2 worked well.
MinObjSize: minimum size (in pixels) of objects. Objects smaller than this value will be removed. Default 1000. Depends on effective pixel size. In our experience, a value corresponding to 3.5 μm^2 worked well.
Note: The displayed default settings in our experience gave very robust results. Small changes to these value should not affect the outcome dramatically.

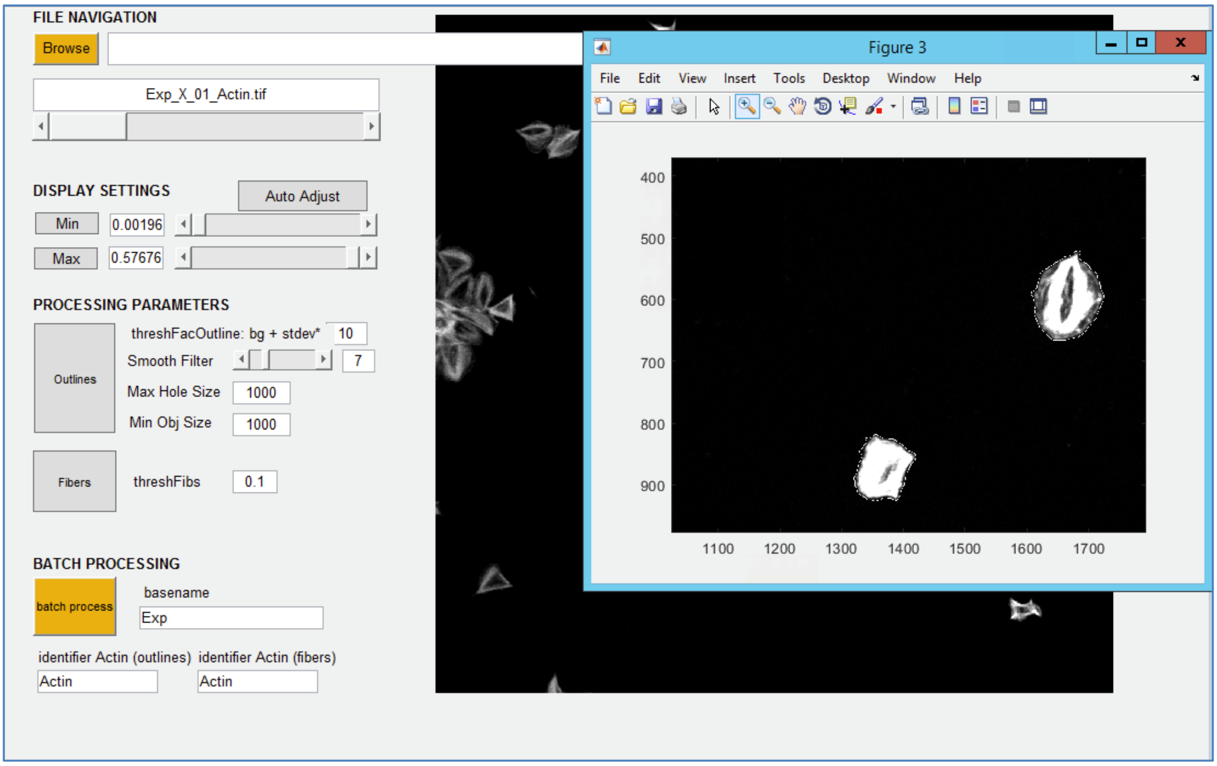

By pressing **Fibers**, check the processing of the fibres in the F-actin image.
threshFibs: threshold value for the detection of fibres. Default: 0.1. Range: 0…1.

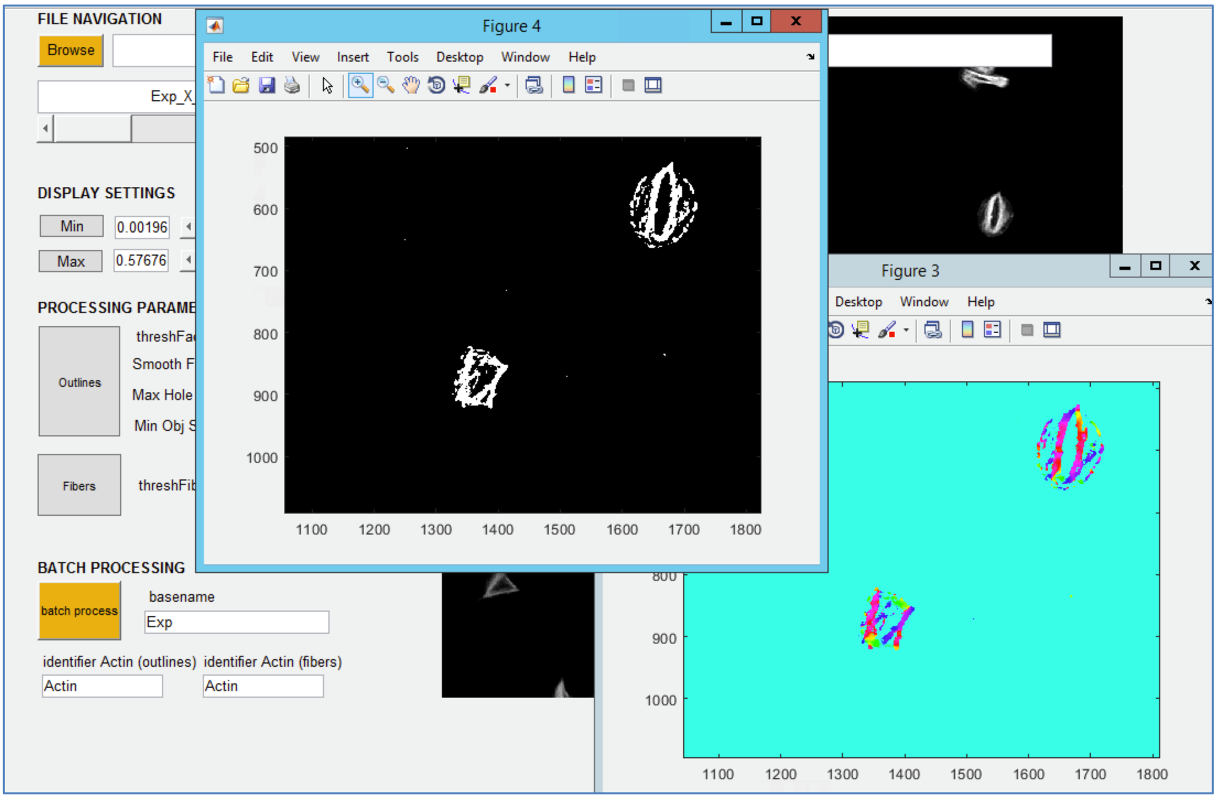

If you are confident with the results, you can process all images in the current folder. Under *BATCH PROCESSING*, define the naming convention:
basename: base name of all images that shall be processed
identifier Actin (outlines): identifier of Actin stain used to create the outlines
identifier Actin (fibers): identifier of Actin stain used to create the fibers
Note: the separate selection of images for the two operations in principle allows you to e.g. use a membrane stain or other stains to define the outline, independent of the f-actin stain. In practice, however, the analysis assumes that both operations are done on f-actin images.

By pressing **batch process** you start with the analysis.

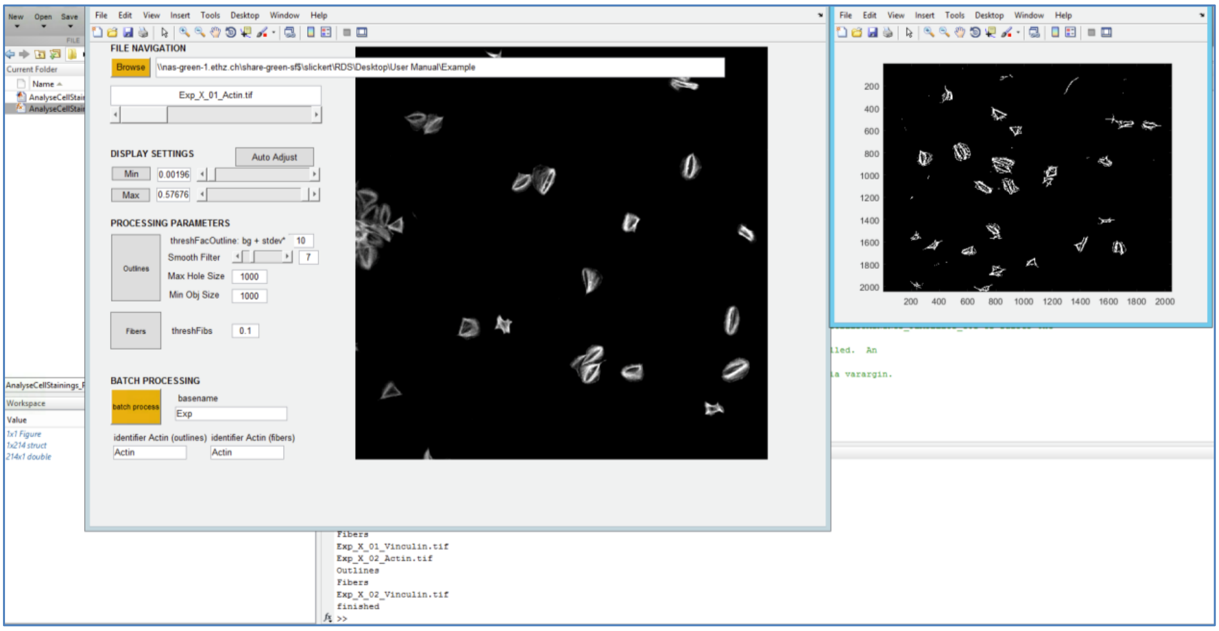

When “finished” in the *Command Window* appears, the analysis is done. The processed images are saved as “*.png”-files in the same folder as the original “*.tiff”-files.

Name	Date modified	Type	Size
Exp_X_01_Actin.tif	12.01.2018 09:52	TIFF image	8 258 KB
Exp_X_01_Actin_Angles.png	23.01.2018 15:17	PNG image	6 600 KB
Exp_X_01_Actin_Filaments.png	23.01.2018 15:17	PNG image	25 KB
Exp_X_01_Actin_Outline.png	23.01.2018 15:17	PNG image	11 KB
Exp_X_01_Vinculin.tif	12.01.2018 09:52	TIFF image	8 258 KB
Exp_X_02_Actin.tif	12.01.2018 09:52	TIFF image	8 258 KB
Exp_X_02_Actin_Angles.png	23.01.2018 15:18	PNG image	7 117 KB
Exp_X_02_Actin_Filaments.png	23.01.2018 15:17	PNG image	22 KB
Exp_X_02_Actin_Outline.png	23.01.2018 15:17	PNG image	13 KB
Exp_X_02_Vinculin.tif	12.01.2018 09:52	TIFF image	8 258 KB

3.4 Edit the cells

Open the folder “02_Edit_Cells” and run the code “Edit_Cells.m” that opens a GUI.

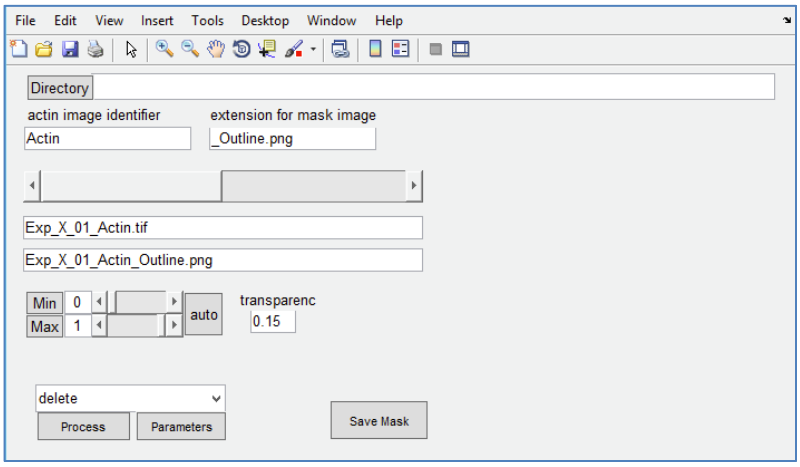

Enter the “Actin image identifier” and leave the “extension for mask image” as it is (“_Outline.png”).

Press **Directory** and select the folder with the processed images.
A new figure appears and displays a transparent color mask (outlines) overlaid with the original f-actin image.
Adjust the contrast of the displayed image by using the sliders or the "Auto" button. The transparency of the overlaid mask can be changed in the field "transparenc"; larger values decrease the transparency.

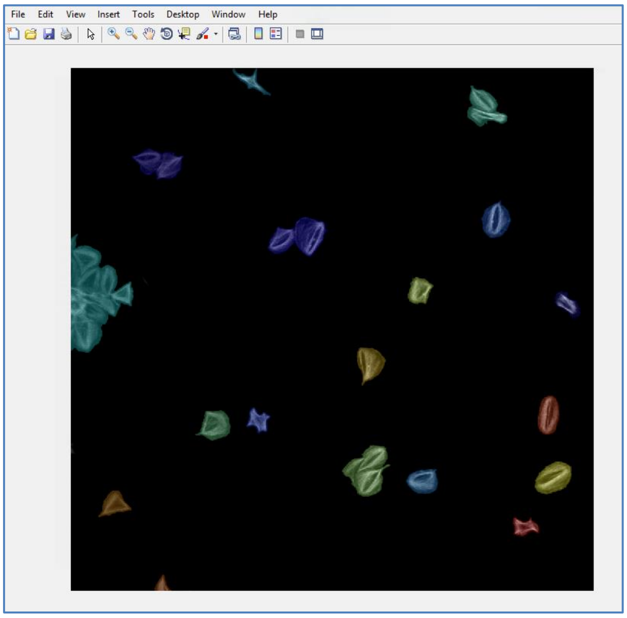

For editing the cells, you can **Process** the following actions: **delete**, **divide**, **cut**, **fill**, **undo last** and **reset.** Some options are controlled under **Parameters**.

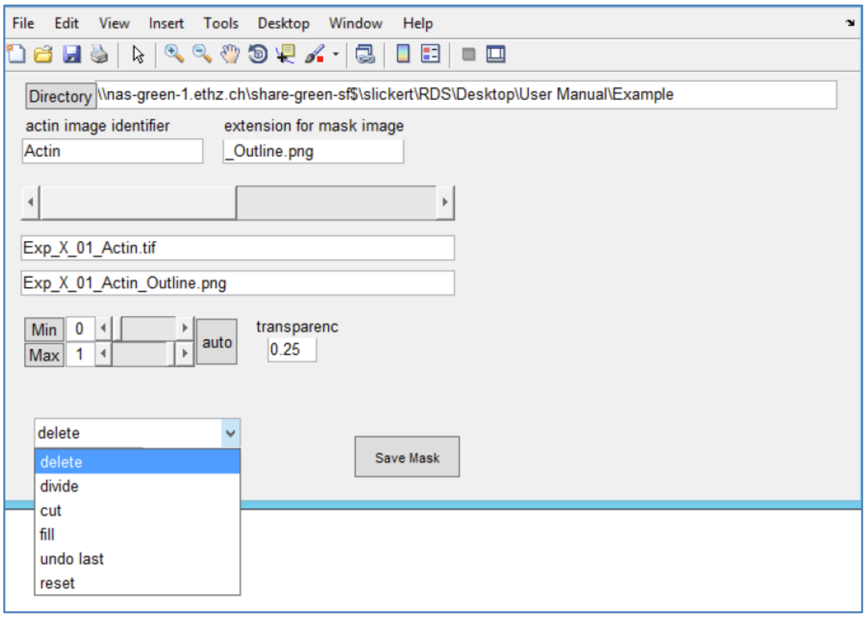

- **Delete:**

By choosing **delete** and then clicking **process**, the following window appears and explains the procedure.

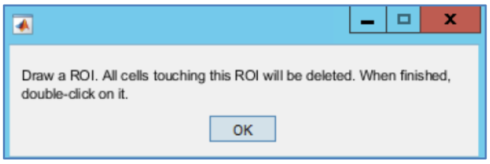

After double-click on the ROI the colored mask disappears.

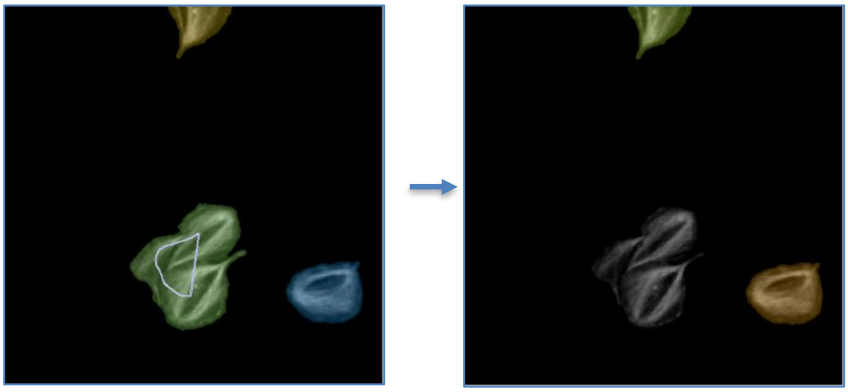

- **Divide:**

By choosing **divide** and then clicking **process**, the following window appears and explains the procedure.

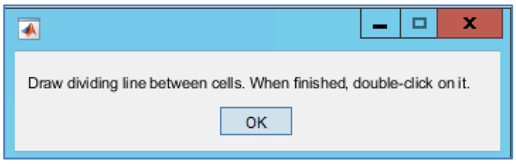

After drawing a dividing line, double-click on the line and the regions are separated, often resulting in a change of color of each separated cell.

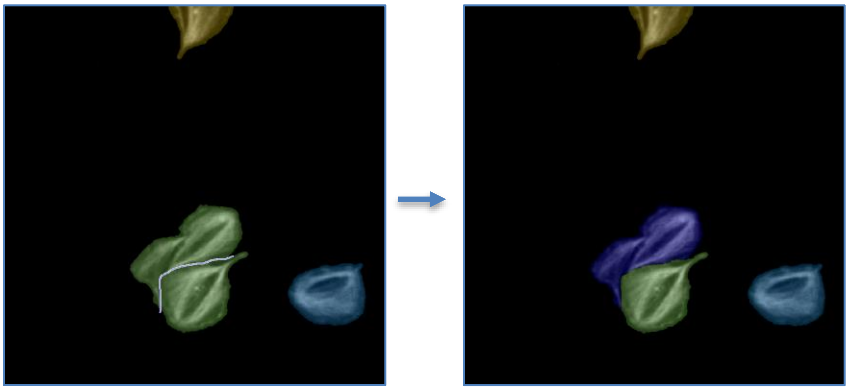

To start a new process, you again have to click **process**.

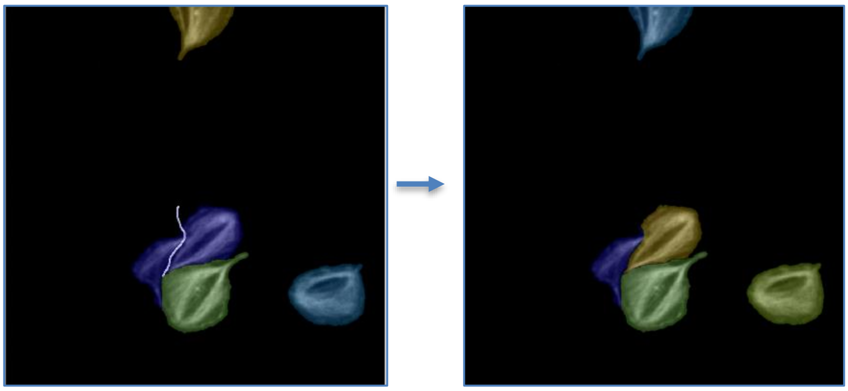

- **Cut:**

This allows you to cut away an unwanted region.

- **Fill:**

Sometimes it could happen, that a region inside of the cell is excluded. Draw a line on the cell and by double-clicking, you can unite the mask.

- **Undo last:**

Here you can undo the last processed step.

- **Reset:**

Before you saved the edited image, you can reset all operations.

By pressing **save** you overwrite the previous "_Outline.png" mask. Please note that the next function ("Process_Cells") requires as a naming convention this "_Outline.png" mask. If you would like to save the unmodified outline masks, please do so before in a separate folder.

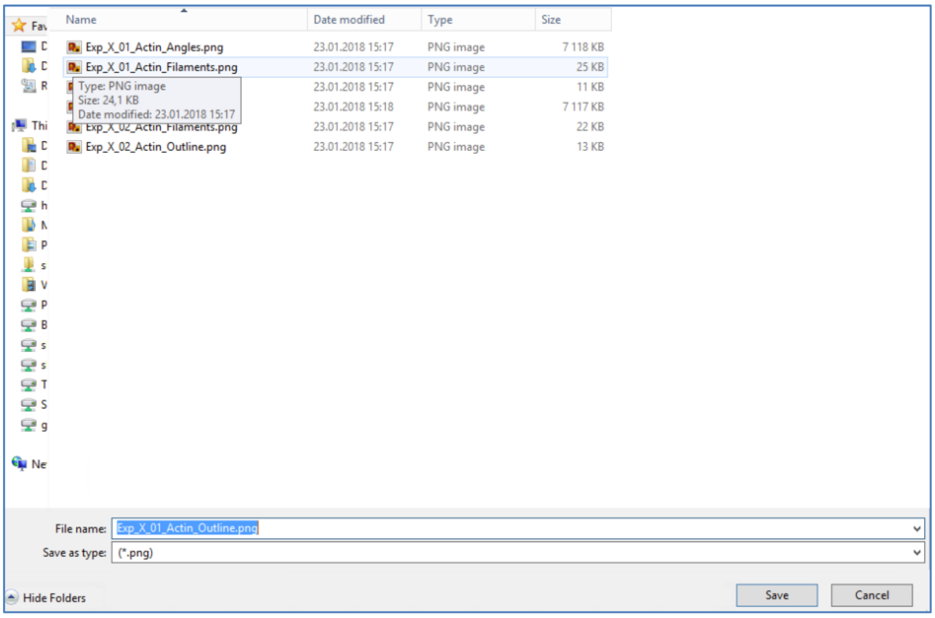

Under **Parameters**, you can change the

- minimum object size (in pixels). All objects smaller than this value will be removed at the end of a processing step.
- Show messages [1=show, 0=suppress]: set this value to zero to suppress the message which is shown before each process step. By setting this value to 0, also the saving proceeds without showing a file save dialog and automatically overwrites the previous mask.

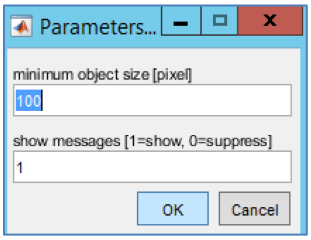

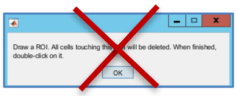

Now, edit each mask in a folder before processing the cells.

Note: This is the only real manual intervention and also the most time-consuming part of the Morphometrics analysis. Normally, it is sufficient to work with delete and divide. Take care, that the seeding density of the platelets is not too high. We normally delete confluent cells or aggregates. Only platelets which are touching each other slightly are divided.

3.5 Process the cells

- Process Cells

Run the code “Process_Cells()” from the command line.
Select the folder with the images and masks.
A window appears and you have to enter the identifier for each channel name.
The 3rd color is not supported at the moment, leave this field empty.
Enter the name tag for the data and the concentration, if you are performing a dose-response curve.

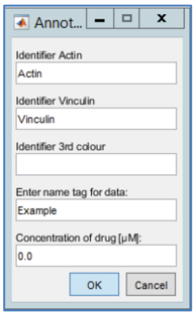

After pressing **OK**, the main part of the image analysis is starting, by analyzing cell by cell. The output is saved into the subfolder “\output”. A figure displays the individual analyzed cells:

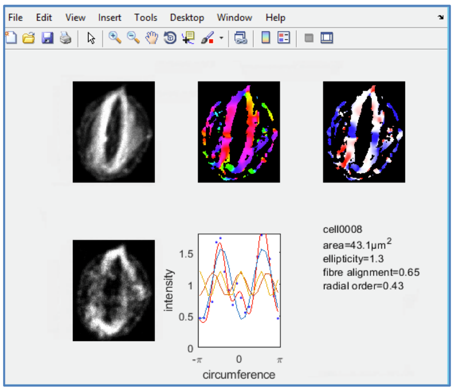

Each figure/cell is saved as a page into a postscript document. You can view this document by any postscript viewer (e.g. GhostView) which is useful to have a look through the individually analyzed cells.
After processing all cells, the results are saved into a "cellstats.mat" variable. You also find individual images in this folder. The code "Process_Cells" at its end also automatically runs the "Display_Morphometrics" command which generates an overview plot with the morphological parameters, and saves this as a figure. For a description of this function, please see the next section.

- Combine Populations

For the meta-analysis of several datasets, the cellstats of individually processed image sets need to be combined. This is done for three purposes:

1) Pooling of data that was collected under the same condition (but e.g. from different donors at different dates)
2) Comparing data that differs in whatever parameters (different donors, adhesion ligands, agonists, etc.)
3) Analysis of dose-response relationships (at different concentrations)

In order to do that, you have to create a new "*.mat"-file with the different desired populations. To do so, run the function "Compare_Populations();". Select every "cellstats.mat"-file you want to compare. If you would like to change the original naming (tag, dose), you can do so at each step.

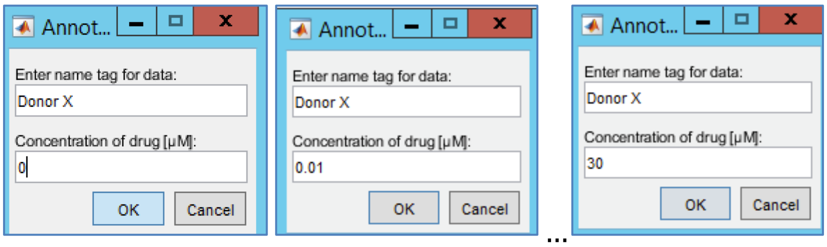

After the last cellstats.mat-file you have added for the comparison, click **cancel** and in the next step **save** your new "*.mat" file under a descriptive name.

3.6 Visualize the cells

- Display_Morphometrics

To visualize and change the settings of the general morphological parameters you can open the program “Display_Morphometrics” in the folder “04_Visualize_Cells”. First you have to open (or drag and drop) your processed “cellstats.mat”-file into the MATLAB *Workspace* (if it is not already there).
Execute the command “Display_Morphometrics(cellstats, *arealimit*);” in the *Command Window* where arealimit needs to be a number and represents the lower threshold (in µm²) above which cells are regarded as spread and analyzed with respect to the cytoskeleton.

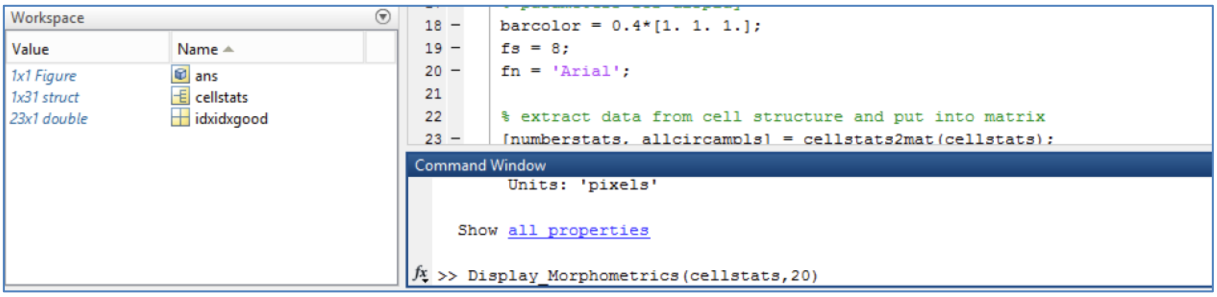

Note: We normally use 20 µm² for the area limit. If you do not enter a value, a default of 5 µm² is used. This threshold does not apply to the analysis of the spreading area which includes cells of all sized.
The execution of the code generates a figure and some output on the *Command Window*.

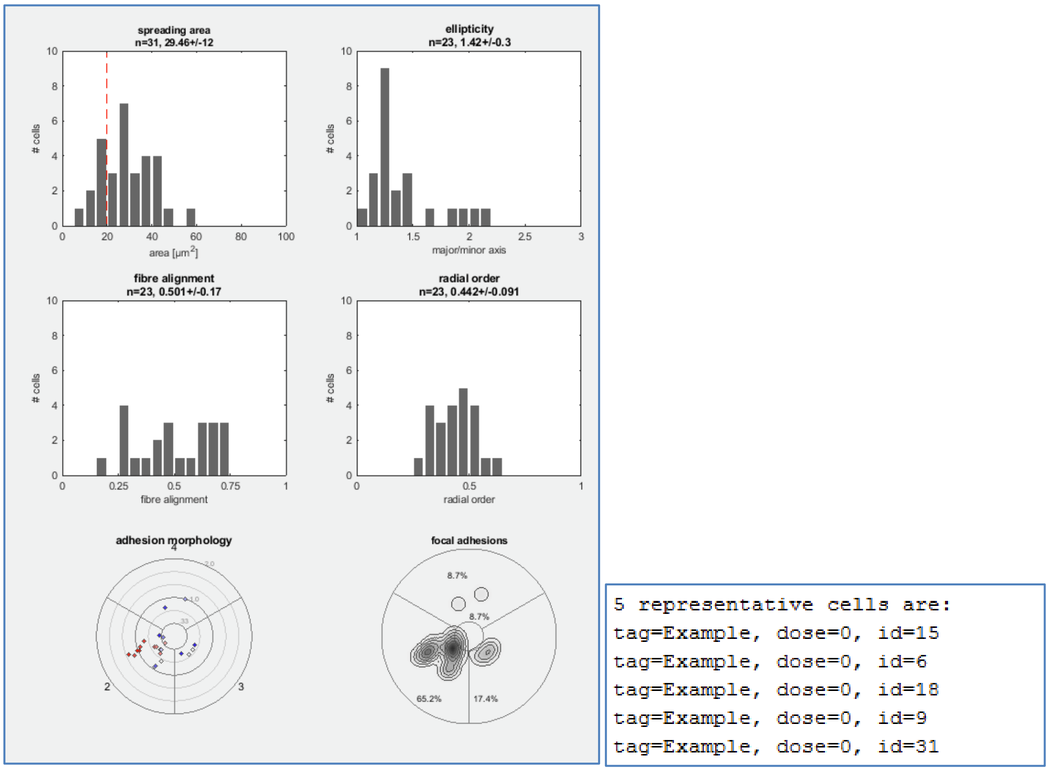


```
5 representative cells are:
tag=Example, dose=0, id=15
tag=Example, dose=0, id=6
tag=Example, dose=0, id=18
tag=Example, dose=0, id=9
tag=Example, dose=0, id=31
```

The figure shows:

- histograms of spreading area (upper left). The red dashed vertical line indicates the *arealimit* that applies to the other analysis in this window.

- Histogram of ellipticity (upper right).
- Histogram of fibre alignment (middle left).
- Histogram of radial order (middle right)
- Adhesion site distribution scatter plot (lower left). The color-code from blue-to-white-to-red indicates the fibre alignment from 0 to 1, clipped at 0.25 and 0.75.
- Contour plot of the adhesion site distribution (lower right). The given percentages indicate the relative numbers of cells in the different sectors.

The Command Window output highlights 5 cells which were located closest to the maximum in the adhesion site distribution plot and thus are deemed to be representative of the prevailing adhesion morphology. Using the identifiers tag, dose, and id, you can look up these cells in the respective "\output" folder, e.g. in the saved postscript file.

- Display_Comparison

You can compare different population using "Display_Comparison". **Load** a file that contains different conditions into the *Workspace*.
With the MATLAB command "Display_Comparison(cellstats, *arealimit*);" you display the comparison in the window. If both different tags and different doses were present in the pooled data, a pop-up window asks you if you would like to compare them by tag or by dose. Make your choice and the processing proceeds.

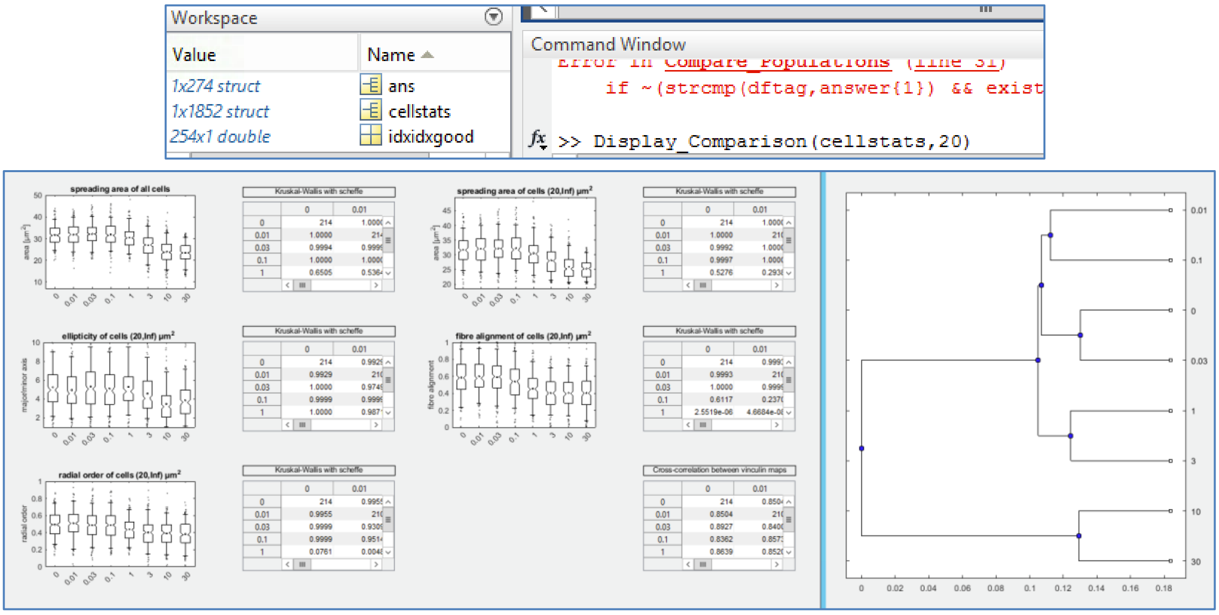

This overview figure depicts the comparison for each parameter between the populations. The tables display the p-values for each comparison based on a Kruskal Wallis test, and on the diagonal the number of cells for this condition. A similarity tree based on the adhesion site distribution plots provides an overview how (dis)similar the different populations are.

- Display_DoseResponse

To create a dose-response curve of a concentration series, use "Display_DoseRespone". Again, you have to create a "*.mat"-file with the "cellstats.mat"-files of the different concentrations. Use therefore again the "Compare_Populations" (see section 3.5). Load this file into the workspace.
With the MATLAB-code "Display_DoseResponse(cellstats, *arealimit*);", you display the curves.

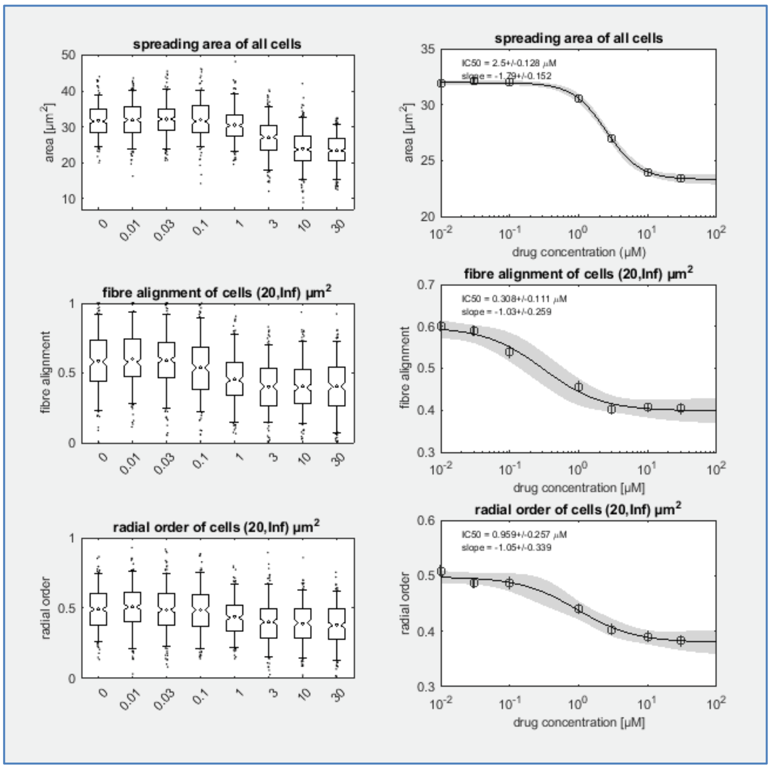

This part of the software also fits and calculates the data by a four parameter logistic curve.

4 Comments

By generating an "*.eps"-file you can export all graphics and manipulate the plots in common vector graphics editors.
To get more familiar with the software, you can play around with the "dummydata"-set, attached to the folder "Workflow_Morphometrics".

For further questions please contact the corresponding author ingmarschoen@rcsi.ie .

Version 1.0, © January 2018.
