# Supplementary figures and images for: Morphometric analysis of spread platelets identifies integrin αIIbβ3-specific contractile phenotype
Source: Sci Rep. 2018 Apr 3;8:5428. doi: 10.1038/s41598-018-23684-w (PMC5882949; doi:10.1038/s41598-018-23684-w)

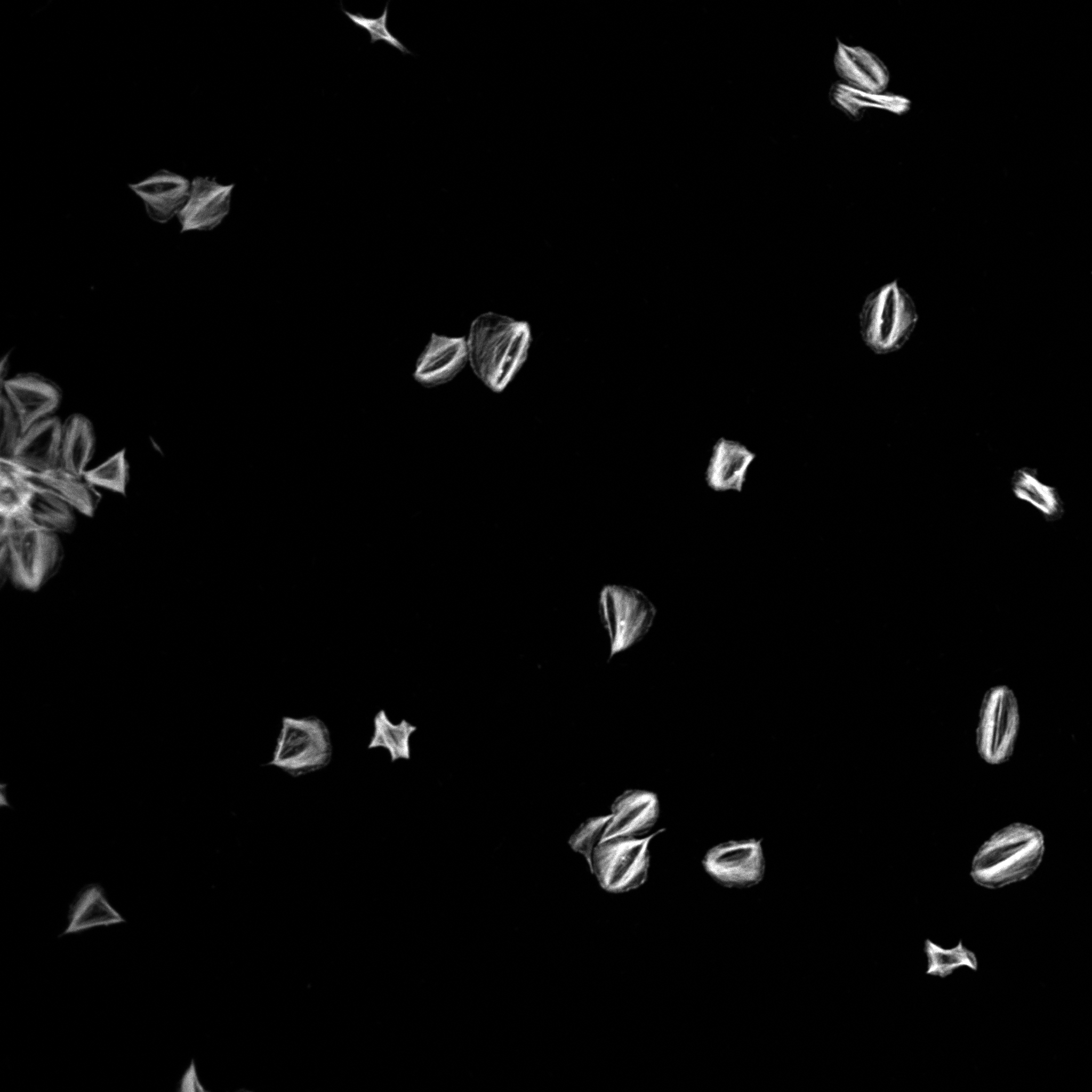

Supplement: Supplementary file 3 — Dataset 1 [file 41598_2018_23684_MOESM3_ESM.zip › Workflow_Morphometrics/Example/Exp_X_01_Actin.tif]

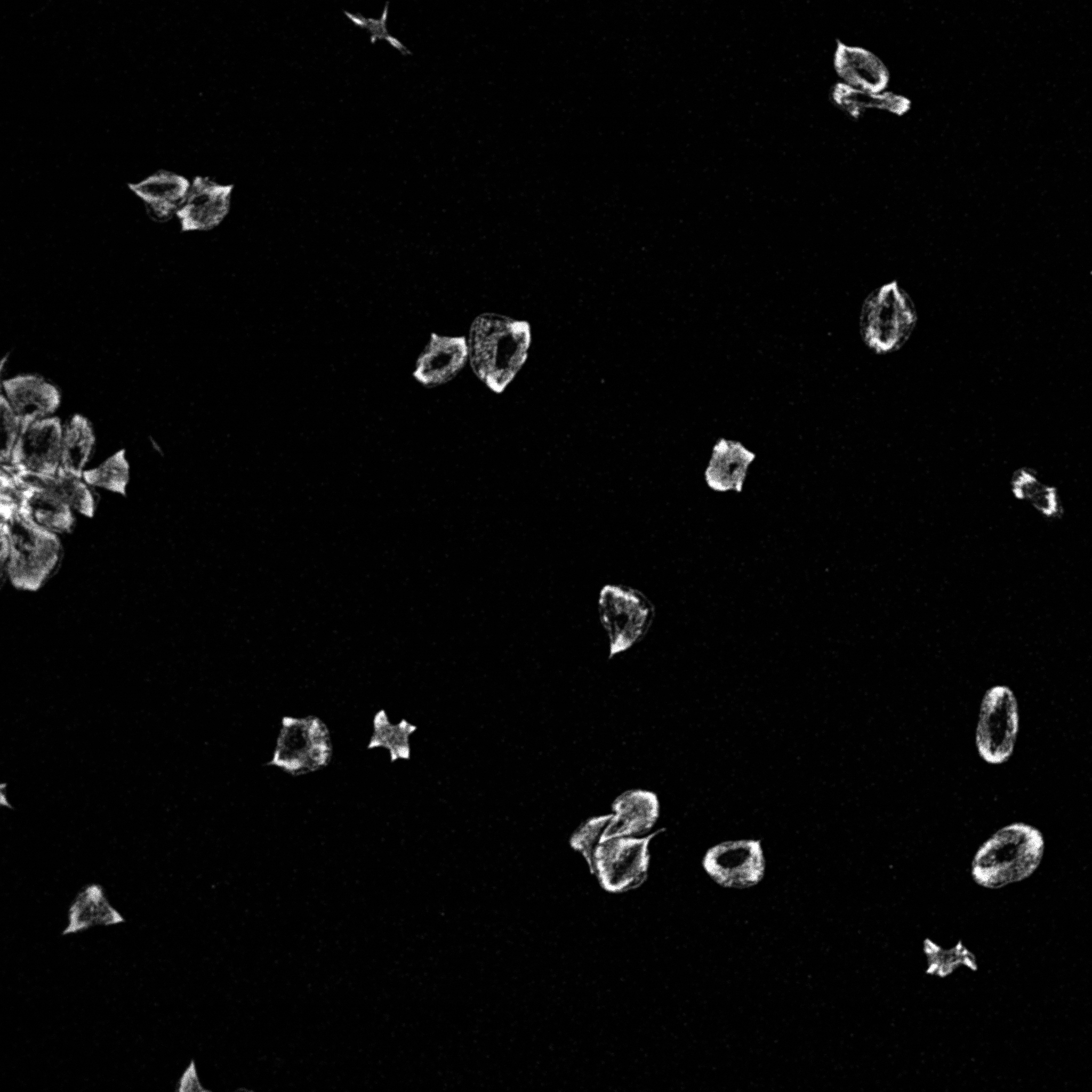

Supplement: Supplementary file 3 — Dataset 1 [file 41598_2018_23684_MOESM3_ESM.zip › Workflow_Morphometrics/Example/Exp_X_01_Vinculin.tif]

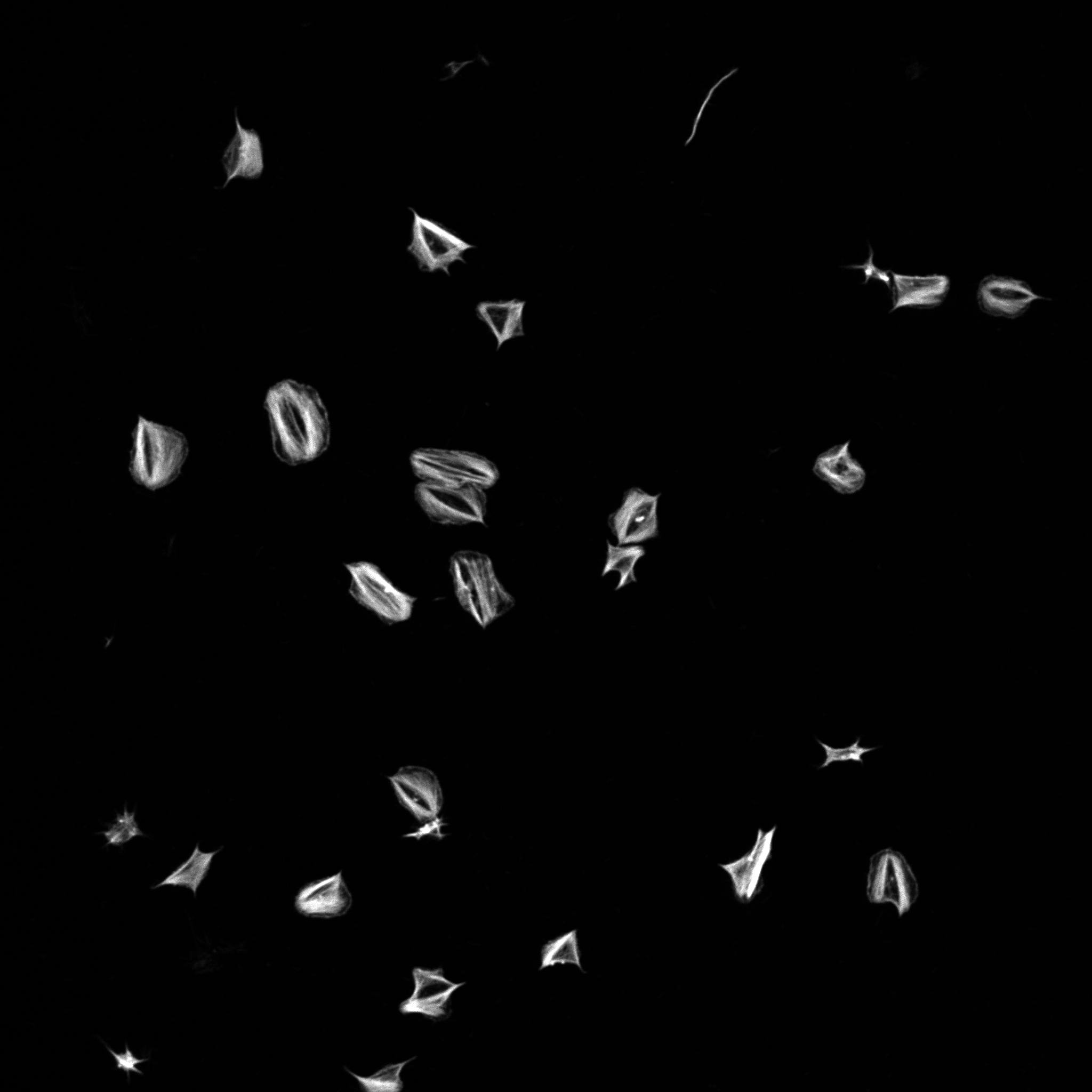

Supplement: Supplementary file 3 — Dataset 1 [file 41598_2018_23684_MOESM3_ESM.zip › Workflow_Morphometrics/Example/Exp_X_02_Actin.tif]

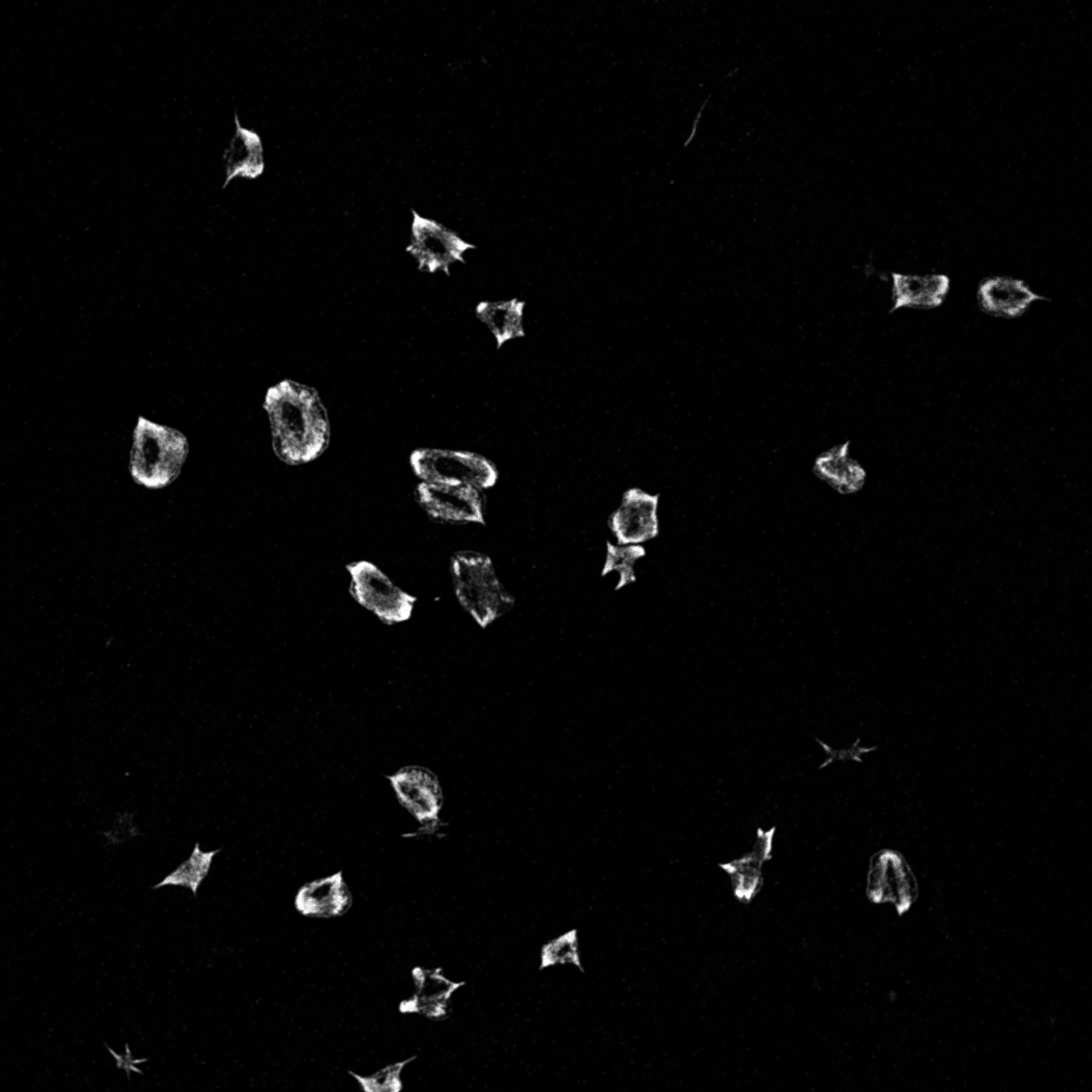

Supplement: Supplementary file 3 — Dataset 1 [file 41598_2018_23684_MOESM3_ESM.zip › Workflow_Morphometrics/Example/Exp_X_02_Vinculin.tif]
